# From mucosal activity to medicinal products: translational evidence for *Akkermansia muciniphila* extracellular vesicles

**DOI:** 10.3389/fimmu.2026.1942618

**Published:** 2026-10-26

**Authors:** Quanli Zhu, Yingchao Liu, Yu Song

**Affiliations:** Department of Gastroenterology, The Second Affiliated Hospital of Zhejiang Chinese Medical University, Hangzhou, Zhejiang, China

**Keywords:** *Akkermansia muciniphila*, Bacterial extracellular vesicles, host-microbe communication, immunomodulation, intestinal barrier, mucosal immunity, nanomedicine, translational evidence

## Abstract

Akkermansia muciniphila extracellular vesicles (AKK-EVs) are being investigated as cell-free treatments for intestinal and metabolic disease, but their therapeutic value depends on what they add beyond whole bacteria, purified microbial components, or alternative carriers. This critical narrative review examines 50 publications available up to 19 July 2026, distinguishing vesicle interventions from studies of associated proteins, whole bacteria, and engineered products. Study-level extraction and domain-based appraisal support the comparison of these preparations. The synthesis addresses product identity, causal attribution, exposure, and therapeutic comparison. Native preparations show activity in epithelial and colitis models, while metabolic findings vary with the preparation and experimental setting. For mucosal disease, the therapeutic aim is sustained functional recovery after disease onset. For liver disease, development depends on establishing whether hepatic benefit persists beyond changes in food intake or body weight and whether it results from intestinal activity or delivery to hepatic cells. Engineered products require evidence that the vesicle carrier improves payload delivery or therapeutic index. Whole-cell human trials and observational measurements of EV-associated proteins provide a clinical basis for comparison, but neither establishes the efficacy or safety of administering isolated AKK-EVs to patients. The evidence supports indication-specific preclinical investigation rather than clinical use. Progression towards human studies requires reproducible batches, disease-relevant functional benefit, route-specific repeat-dose safety, and an advantage over a justified active comparator.

## Graphical Abstract

**Figure:**
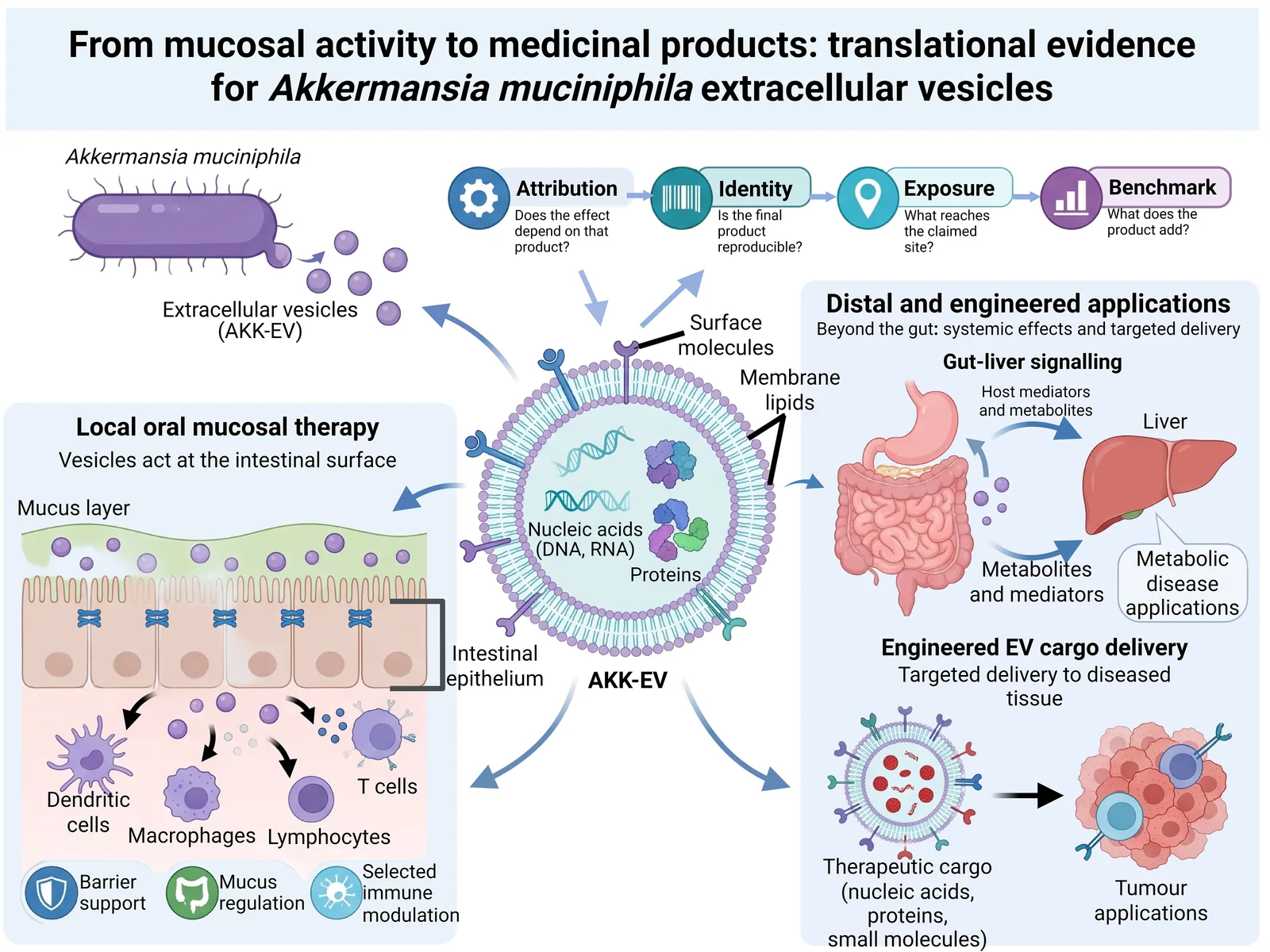
Therapeutic applications of Akkermansia muciniphila extracellular vesicles. The schematic links vesicle-associated molecules to three lines of investigation: local mucosal activity, gut–liver signalling through host mediators and metabolites, and engineered cargo delivery. Identity, Attribution, Exposure, and Benchmark organise the evaluation of each preparation. The pathways illustrate proposed applications whose efficacy and safety require product-specific testing. Created in BioRender. Song, Y. (2026) https://BioRender.com/u99xms0.

## 1 Introduction

The clinical interest in Akkermansia muciniphila lies in whether its interaction with the intestinal mucosa can be turned into a reproducible treatment (1). The bacterium occupies a mucus-associated niche at the interface between the intestinal contents and the host (2). Experimental studies link A. muciniphila to epithelial and metabolic responses in obesity and to intestinal adaptive immunity (3, 4). These findings motivate treatments that restore mucosal function or modify gut-derived signals contributing to metabolic disease.

Extracellular vesicles offer a way to administer bacterial products without delivering replicating organisms. Their membranes carry proteins, lipids, and other molecules that could act without sustained colonisation. For treatment, the potential advantage lies in controlling dose and timing while retaining useful bacterial activity. A vesicle formulation could also protect an introduced therapeutic payload. Direct comparison with whole bacteria or a purified active component is needed to establish whether the vesicle format improves efficacy, tolerability, or delivery.

Microbiota-derived vesicles participate in host–microbe communication and influence immune responses (5, 6). The value of those responses depends on the disease being treated. An immune response that supports tumour treatment could aggravate inflamed intestinal tissue. For hepatology, the appeal extends beyond delivery to hepatocytes. An oral preparation might benefit liver disease through intestinal activity, altered metabolism, or reduced inflammatory signalling. These possibilities call for different measures of exposure and therapeutic benefit.

Akkermansia muciniphila extracellular vesicle (AKK-EV) studies now encompass colitis, obesity, liver injury, cancer, and neurological disease. Across these settings, the term EV refers to preparations with different origins, compositions, and intended functions. A native preparation for mucosal repair, a vesicle-associated signal during drug treatment, and an engineered carrier address different therapeutic questions. Interpreting their results requires determining which activity is attributable to the preparation and whether that activity improves an outcome relevant to the intended disease.

Recent reviews describe AKK-EV biology and its metabolic and therapeutic applications (7, 8). Here, we examine what the vesicle format could add to treatment and which experiments would establish that value. We connect disease-specific findings to the proposed site of action, relevant comparators, and outcomes that could support further clinical development. Throughout, AKK-EV, AKK-derived EV, and AKK-derived extracellular vesicle refer to preparations derived from A. muciniphila. We use outer-membrane vesicle (OMV) where that origin is supported.

## 2 Methods

### 2.1 Literature search and study selection

We conducted a critical narrative review using PubMed/MEDLINE, with the core search performed on 22 July 2026 and publication eligibility restricted to studies published through 19 July 2026. The search combined two title/abstract concept groups with AND: (“Akkermansia muciniphila” OR Akkermansia) and (“extracellular vesicle” OR “extracellular vesicles” OR “outer membrane vesicle” OR “outer membrane vesicles” OR OMV OR OMVs OR “membrane vesicle” OR “membrane vesicles”). Terms within each group were combined with OR. Supplementary searches added disease terms, including barrier, colitis, inflammation, obesity, and liver, as well as engineering and delivery terms. Reference lists, publisher pages, and identifier searches provided additional reports. **Supplementary Table S1** gives the complete recorded queries, including their [tiab] field tags, and the targeted ClinicalTrials.gov query.

We included original studies reporting the composition, biological activity, mechanism, exposure, or safety of preparations derived from A. muciniphila, including native or enriched EVs, engineered vesicles, mechanically generated vesicle-like preparations, and EV-associated components. Cell, animal, and human observational studies were eligible and classified by the material examined. Reviews, editorials, and records without original study data, reports unrelated to AKK-derived vesicles or their components, and publications appearing after the cutoff were excluded from the study-level synthesis. Whole-cell-only interventions and studies of other bacterial EVs or synthetic carriers informed background or comparator choice. Duplicate versions and corrections were considered with the parent report. Final publications were preferred; retained abstracts, publisher previews, and linked preprints were identified by source type, with unavailable methods recorded explicitly. Two reviewers independently screened the records and assessed the available reports against the eligibility criteria. They agreed on the final inclusion and exclusion decisions.

The 107 core records comprised 47 inclusions, 59 exclusions, and one unresolved record; three additional-source reports brought the synthesis to 50 publications. **Supplementary Table S1** and the screening workbook document source reconciliation and study decisions (9).

### 2.2 Data extraction, appraisal, and synthesis

Two reviewers independently extracted study model, strain, culture conditions, vesicle preparation and characterisation, dose, route, treatment schedule, sample size, controls, outcomes, mechanistic tests, exposure, and safety. Extracted information was checked against the source text and figures. Missing or inconsistent details were recorded in the extraction tables. Stable identifiers link the study findings, preparation details, and experimental features in **Supplementary Tables S2**-**S4**.

Two reviewers independently assessed animal EV intervention studies using the ten SYRCLE risk-of-bias domains (10) and agreed on the final judgments. Each domain was rated low, high, or unclear; inadequate reporting was rated unclear. The animal-study tool did not apply to cell-only studies, human observational measurements, or purified-protein interventions. **Supplementary Table S5** presents the domain judgments. No overall quality score or chance-corrected agreement statistic was calculated.

Findings were synthesised by preparation, administration route, and clinical question: mucosal treatment, gut–liver effects, or therapeutic cargo delivery. **Tables 1**, **2** relate human whole-cell comparators and preclinical findings to treatment decisions. Differences in preparations, models, dose units, and outcomes precluded quantitative pooling.

**Table 1:** Human whole-cell Akkermansia trials: implications for EV development.

Study and population	Whole-cell intervention	Clinical finding	Relevance to EV development
Exploratory trial; 40 enrolled, 32 completed (28)	Live or pasteurised A. muciniphila; 10^10^ bacteria daily for 3 months	Tolerability and selected metabolic improvements were reported with pasteurised bacteria.	Provides a whole-cell comparator and exploratory metabolic outcomes.
Metabolic syndrome; 142 adults (29)	Pasteurised A. muciniphila MucT; 3 × 10^10^ cells daily for 4 months	No difference from placebo in the primary intention-to-treat Matsuda insulin-sensitivity endpoint.	Population and primary outcome should be prespecified; subgroup findings remain exploratory.
Diet-led weight loss; 90 adults (30)	Pasteurised A. muciniphila MucT or placebo during 24-week maintenance after an 8-week low-energy diet	Weight regain: 1.2 kg ± 0.7 kg versus 3.2 kg ± 0.4 kg; *p* = 0.012. No treatment-related serious adverse events reported.	Identifies weight-loss maintenance as a distinct clinical use to investigate.

All interventions in this table used whole bacteria. The results do not provide an isolated-EV dose, efficacy estimate, or safety profile.

**Table 2:** AKK-EV disease models, therapeutic findings, and further experiments.

Clinical question and models	Preparation and route	Observed preclinical result	Decisive next test
Mucosal recovery: DSS colitis; HFD barrier injury; Caco-2 and LS174T cells	Native EV/OMV preparations; oral dosing or cell exposure	Reduced colitis injury and improved permeability or mucus-related responses in distinct models. Additional OMV experiments include colitis and combination tumour therapy.	Treat established disease; compare process and whole-cell controls. Confirm functional recovery before considering symptom and endoscopic outcomes.
Metabolic benefit: diet-induced obesity and glucose dysregulation	Native EV/OMV preparations; product-specific oral regimens	Lower weight gain or adiposity in several models. One small EV experiment showed no glucose benefit; metabolic outcomes varied across experiments.	Use pair-fed and whole-cell controls. Establish benefit beyond intake reduction; select insulin sensitivity or weight maintenance for the intended population.
Hepatic benefit: HFD/CCl4 injury; diet-induced MASH; LX-2 cells	Culture-derived or PEG-enriched preparations; oral dosing in mice	Liver-injury and fibrosis-related responses in toxin-assisted models; lower liver lipid accumulation in a MASH model.	Confirm a purified product in established diet-induced disease. Assess histology and fibrosis alongside intake, weight and intestinal function.
Colorectal tumour control under chronic stress: AOM/DSS model	Culture-derived OMVs; daily oral dosing	Lower tumour burden; Fetuin-A replacement attenuated benefit in a related report.	Test dependence on the stress-related mechanism and compare an active treatment before defining a clinical population.
HCC delivery and immunotherapy: mouse and patient-derived models	EV-coated PD-L1 siRNA–MOF composite; intravenous dosing plus ultrasound	Target silencing, pyroptosis and immune changes accompanied tumour suppression.	Use component controls and relevant treatment. Measure tumour versus nontumour liver exposure, target engagement and repeat-dose toxicity.
Cargo delivery in other tumour models: 4T1 breast and CT26 colorectal tumours; oral RNA-delivery models	Intravenous loaded OMVs or targeted AKK–E. coli hybrids; separate oral RNA-delivery platform	Copper-pathway- or PD-L1-directed constructs showed tumour activity. Carrier, payload, and hybrid-membrane effects remain product-specific.	Compare matched payload and carrier controls. Measure systemic inflammatory tolerance for injection and mucosal integrity for oral delivery.

Study sources are cited in Sections 4.1, 4.3, 4.4, 5.3, and 5.4; full study-level records are in the supplement. Observed effects are preclinical. The final column gives proposed comparisons and endpoints, which do not demonstrate patient benefit. Obesity, toxin-assisted liver injury, and stress-associated colorectal tumour reports include provisional related pairs.

HFD, high-fat diet; DSS, dextran sulphate sodium; CCl4, carbon tetrachloride; MASH, metabolic dysfunction-associated steatohepatitis; MOF, metal–organic framework; AOM, azoxymethane; PEG, polyethylene glycol; HCC, hepatocellular carcinoma; PD-L1, programmed death-ligand 1; siRNA, small interfering RNA.

Identity addresses reproducible composition, production, and dosing; Attribution separates preparation activity from mediator necessity; Exposure distinguishes label, particles, cargo, and host responses; Benchmark asks what the product adds to a relevant treatment. Separate preparations were not combined into a causal pathway.

These questions adapt established principles to heterogeneous products: GRADE addresses outcome-level certainty, Hill informs causal interpretation (11, 12), ARRIVE guides animal-study reporting, and MISEV guides EV characterisation and functional testing (13, 14). Their product-level organisation connects composition, causal attribution, target-site exposure, and therapeutic comparison. The synthesis is qualitative and does not assign numerical scores or rank clinical indications.

**Box 1** defines the questions, **Box 2** applies them to mucosal experiments, and **Box 3** proposes criteria before human testing.

BOX 1 **Product identity, causal attribution, exposure, and benchmark.**

Identity: Can the preparation and dose be reproduced?

Attribution: Which component is responsible for activity, and is vesicular packaging necessary?

Exposure: What reaches the site of action: intact particles, released cargo, or a host-derived signal?

Benchmark: What does the preparation add to a whole-cell product, purified component, alternative carrier, or relevant treatment?

Box 2 **Confirmatory testing of oral AKK-EVs in mucosal disease.**

Use a specified strain bank, manufacturing process, formulation, and dose. Carry uninoculated medium through the same process and compare the final preparation with EV-depleted or disrupted material and an appropriate whole-cell product or active treatment. Start treatment after disease is established. Prespecify a functional permeability or mucosal-injury endpoint, justify sample size, randomise allocation, and blind assessment. Measure local recovery, inflammation, and clinical benefit. Repeat the experiment with independently manufactured lots. Where a mediator is proposed, test its necessity and rescue. **Box 3** adds manufacturing and safety criteria before human exposure. **Figure 2** places these tests along the oral route.

Box 3 **Preclinical criteria for first-in-human studies.**

Reproducibility: show prespecified identity, purity, integrity, and potency ranges in at least three independently manufactured lots, with blinded replication of a functional disease effect. Three lots are an author-proposed planning minimum, not a validated threshold.

Safety: justify the exposure margin and demonstrate acceptable local or systemic findings for the final formulation, intended route, and repeat-dose schedule. Unexplained inflammation, viable-cell contamination, or inconsistent potency requires resolution before progression.

Clinical relevance: demonstrate benefit after disease onset using a prespecified functional endpoint; assess liver injury and fibrosis alongside food intake and body weight in hepatic programmes.

Comparison: include process controls and an active comparator at justified exposures. Engineered products additionally need payload and carrier controls. Reconsider the EV format when it provides no meaningful advantage.

Human-study entry: review the manufacturing, efficacy, exposure, and safety evidence together for the selected product, population, and indication.

Sensitivity analysis compared 50 separate reports with 46 provisional groups formed from four related pairs identified in **Supplementary Table S2**. ST37 reused ST11 tissues; the other three links remain provisional. Grouping reduces the apparent amount of independent corroboration without combining separate preparations into a causal pathway. These groups are not confirmed independent cohorts, and their number does not establish clinical priority.

## 3 Vesicle biology and product development

The rationale for vesicle therapy depends on an advantage of the preparation itself. Separating activity from replication may allow a controlled dose, while membrane packaging may protect cargo or alter its presentation to host cells. The same membrane can also carry inflammatory components. These properties make the whole-cell product and the purified active molecule important comparators throughout development.

### 3.1 Vesicle biogenesis and bioactive components

Gram-negative bacterial EVs can form by outer-membrane blebbing or through lysis-associated processes (15, 16). Their origin affects cargo composition, accompanying material, and reproducibility. When the origin or purity remains unresolved, an EV-enriched preparation is a more precise description than a homogeneous population of OMVs.

Centrifugation, filtration, and electron microscopy are common in the retained studies. A recovered pellet can contain vesicles alongside soluble proteins, medium constituents, and membrane fragments. Complementary measurements of particles, composition, and integrity are therefore needed to define the administered material. Processing uninoculated medium in parallel and checking for viable producer cells would identify two important sources of unintended activity.

Several *A. muciniphila* components have activity outside a demonstrated vesicle-dependent mechanism. Amuc_1100 has barrier-related and metabolic effects, while P9 stimulates glucagon-like peptide-1 release (17, 18). A defined phospholipid and lipooligosaccharide influence immune signalling (19, 20). Amuc_1409 supports intestinal stem-cell regulation in experimental systems (21). These observations make purified components biologically relevant comparators. Fractionation, membrane disruption, and selective cargo degradation can locate an activity; loss-of-function and rescue experiments can then test its contribution to treatment.

### 3.2 Strain selection, production, and comparability

Strain background and culture conditions can change the activity being manufactured. Clade-dependent EV effects on bacterial competition and (Immunoglobulin A) IgA-associated niche occupancy provide direct evidence of functional variation (22). Mucin depletion changes bacterial gene expression and whole-cell activity, although its effect on purified EV cargo requires separate measurement (23). Media, conditioning, harvest, and purification therefore need to be specified before results can be compared between laboratories.

Strain banks should retain accession, genome-based identity, clade, purity, and passage history. Fermentation records should specify medium and mucin source, anaerobic conditions, pH, temperature, and growth duration. Harvest criteria should include growth phase, biomass, viability, and indicators of lysis. Cell-bank and comparability principles in International Council for Harmonisation of Technical Requirements for Pharmaceuticals for Human Use (ICH) Q5D and Q5E provide a useful manufacturing analogy (24, 25). Their application to AKK-EVs requires product-specific acceptance limits.

Interlaboratory comparison is most informative when laboratories receive blinded aliquots of a shared reference lot and use a common functional assay. Particle number, protein, integrity, impurities, and storage history should accompany that assay. Production variables can then be changed individually while strain, purification, and testing remain fixed. Agreement in size is less informative than agreement in the intended biological activity.

Exposure assays should identify the material responsible for the signal. A fluorescent label may detach, transfer to host membranes, or persist after particle degradation. Membrane and cargo measurements, removal of unbound label, and recovery of associated material from tissue would strengthen a delivery claim. Local mucosal contact may be sufficient for an oral barrier treatment; a carrier intended for a distant organ requires evidence that its active payload reaches the relevant cells.

### 3.3 Human whole-cell studies and EV development

Whole-cell products provide the closest human clinical comparison. Live bacteria can respond to the intestinal environment and release active substances, whereas pasteurisation removes replication while retaining selected components. The ISAPP postbiotic definition encompasses preparations of inanimate microorganisms and/or their components that confer a health benefit (26). That category does not establish equivalence among whole cells, purified components, and EV preparations. **Figure 1** distinguishes the products that need separate evaluation.

**Figure 1:**
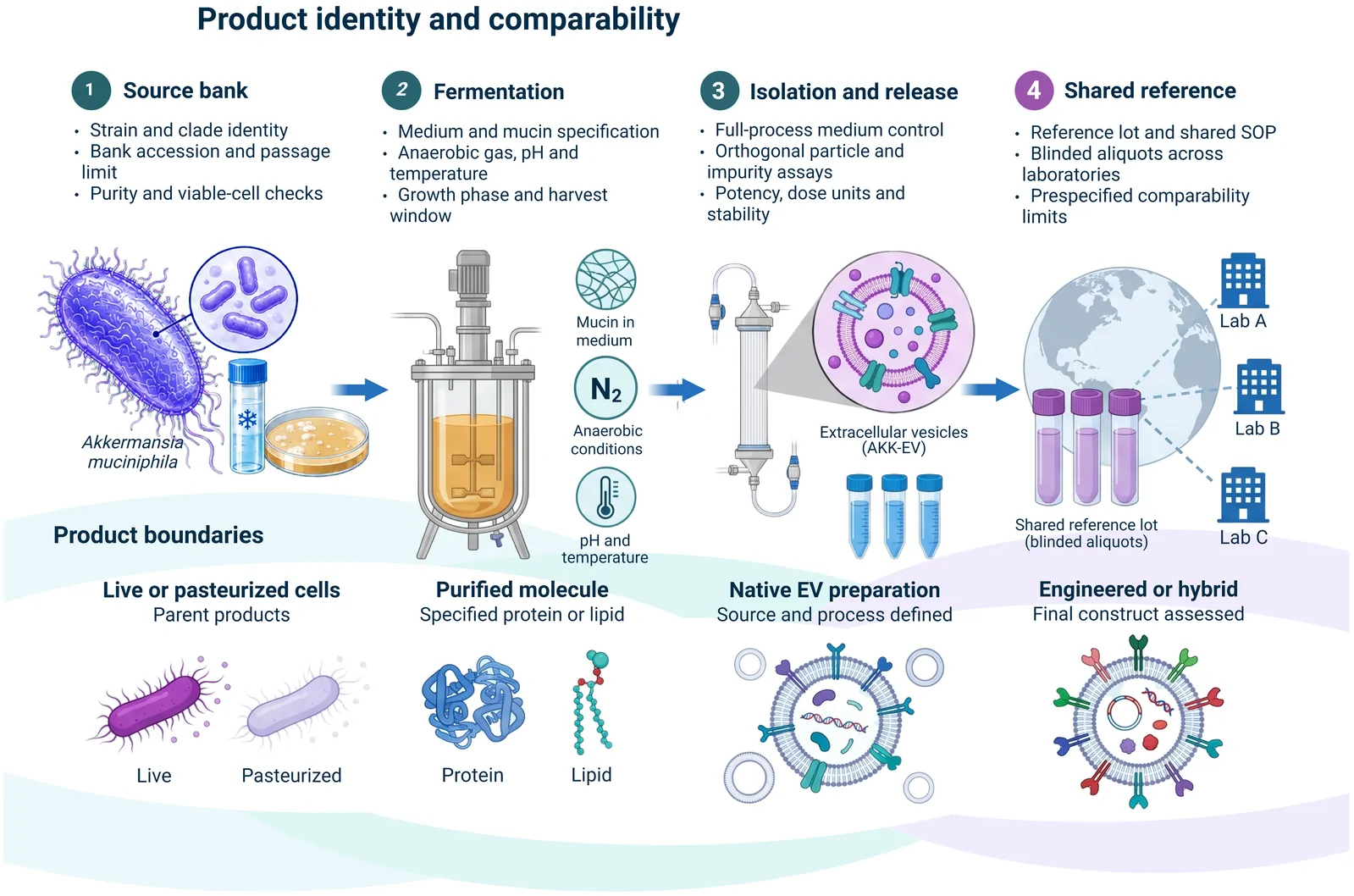
Product identity and comparability. Four stages connect the producer strain to a preparation that can be compared across laboratories. Source-bank records specify strain, clade, accession, passage limits, and purity. Fermentation records define the medium, mucin, anaerobic conditions, pH, temperature, and harvest window. Isolation and release testing combine process controls with particle, impurity, potency, dose, and stability measurements. A shared reference lot, standard operating procedures, and blinded aliquots support interlaboratory comparison. Bottom: Whole cells, purified molecules, native EV preparations, and engineered or hybrid products, each of which requires its own specifications. Created in BioRender. Song, Y. (2026) https://BioRender.com/2umudwz.

**Table 1** illustrates why clinical development needs a defined population and endpoint. An exploratory metabolic signal, a negative primary insulin-sensitivity endpoint, and benefit during weight-loss maintenance address different clinical questions. The trials inform comparator choice and study design for AKK-derived products. Human plasma measurements of EV-associated Amuc_1473 provide observational evidence, not evidence of an administered EV treatment (27). No human intervention with isolated AKK-EVs was identified in the literature retained through 19 July 2026. The whole-cell trials are summarised in **Table 1** (28–30).

## 4 Therapeutic evidence across disease models

The preclinical evidence is organised by the clinical problem being addressed. **Table 2** brings the principal experiments with the next useful comparison and endpoint. Related reports are grouped where appropriate. Disease categories overlap, so table rows cannot be summed as independent replications.

### 4.1 Epithelial barrier function and experimental colitis

The epithelial studies connect AKK-derived preparations to functions relevant to mucosal recovery. Chelakkot et al. reported permeability and junctional responses in epithelial systems and high-fat-diet mice (31). Whole bacteria and EVs produced different Toll-like receptor and junctional responses (32). In DSS colitis, EV preparations reduced injury and inflammation, with mucus-associated responses in some models (33–35). Other studies examined LPS-challenged human colorectal adenocarcinoma (Caco-2) cells, dose-specific junctional responses, and mucin secretion in indomethacin-exposed LS174T cells (36–38). An additional Caco-2 experiment examined bacterial derivatives during exposure to Clostridioides difficile toxin-containing supernatant; the accessible abstract does not establish a treatment effect of isolated EVs in vivo (39). Functional permeability and mucus recovery are useful confirmatory outcomes, while a single junctional marker gives a narrower readout.

For ulcerative colitis, the clinical aim would be improved mucosal recovery alongside established therapy. Symptoms and endoscopic healing are important outcomes, with inflammatory biomarkers providing supporting information (40). The additional colitis experiments broaden the evidence but do not establish comparable effects across strains, purification methods, and dose units. Treatment of established disease, blinded functional assessment, and replication with independently manufactured lots remain useful next tests. Confirmation in patient-derived mucosal systems would then help assess relevance to the intended population. An accessible intestinal site makes these questions practical to investigate; it does not demonstrate clinical superiority over other routes. **Figure 2** summarises the proposed testing sequence for oral delivery and local mucosal activity.

**Figure 2:**
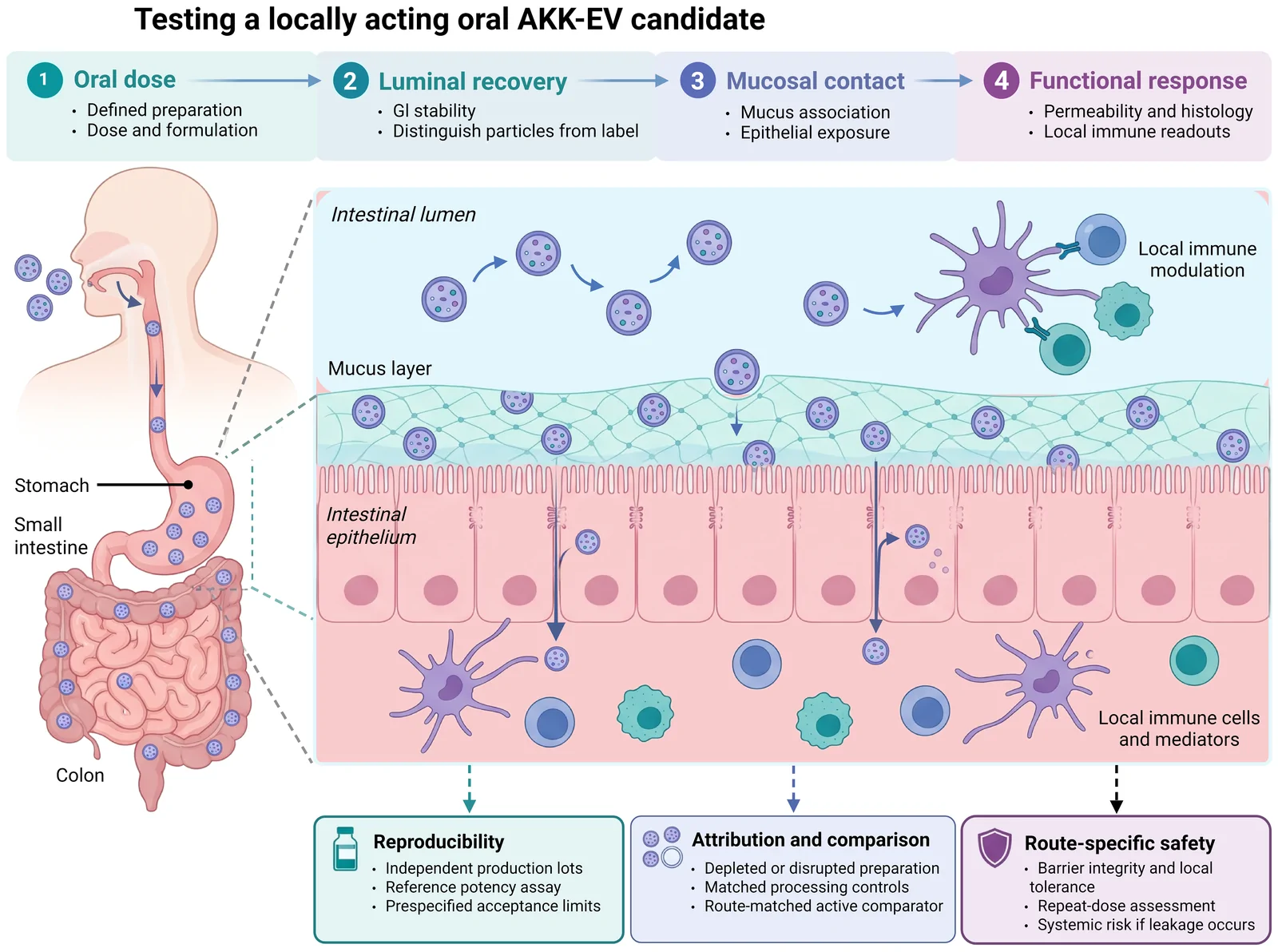
Oral delivery and mucosal activity of AKK-EVs. The upper sequence identifies four experimental stages: administration of a defined preparation, recovery from the intestinal lumen, mucosal contact, and a functional response. The intestinal schematic illustrates candidate interactions with mucus, epithelial cells, and local immune cells. Arrows connect proposed transit and interaction steps that require verification for the administered preparation. Functional testing should include permeability, histology, and local immune readouts. Bottom: Independent-lot replication, preparation and processing controls, an active comparator, and route-specific assessment of barrier integrity, local tolerance, and repeated exposure. Particle recovery should be distinguished from persistence of a fluorescent label. Created in BioRender. Song, Y. (2026) https://BioRender.com/ebbqlp2.

### 4.2 Innate immune modulation in inflammatory disease

Immune readouts should be interpreted in relation to the treatment goal. Studies have measured microRNA responses in human dendritic cells and endocannabinoid-system transcription in vitro (41, 42). Other work reported reduced smoking-related prostate inflammation and fibrosis; its intravenous tracing experiment used a different route from oral treatment (43). A polymyxin B-conjugated OMV preparation was investigated in asthma (44). In atopic dermatitis, miR-21-5p manipulation and a TLR4 reporter supported mediator involvement, but the administered preparation was not tested after miRNA depletion (45). Reduced (NLR family pyrin domain containing 3) NLRP3-related readouts in aged mice exposed to sevoflurane were reported without a perturbation test of that pathway (46). These results identify possible mechanisms and disease applications. Confirmatory work needs to connect the selected immune response to functional benefit and tissue tolerance. **Figure 3** separates these immune observations by cell type or signalling pathway.

**Figure 3:**
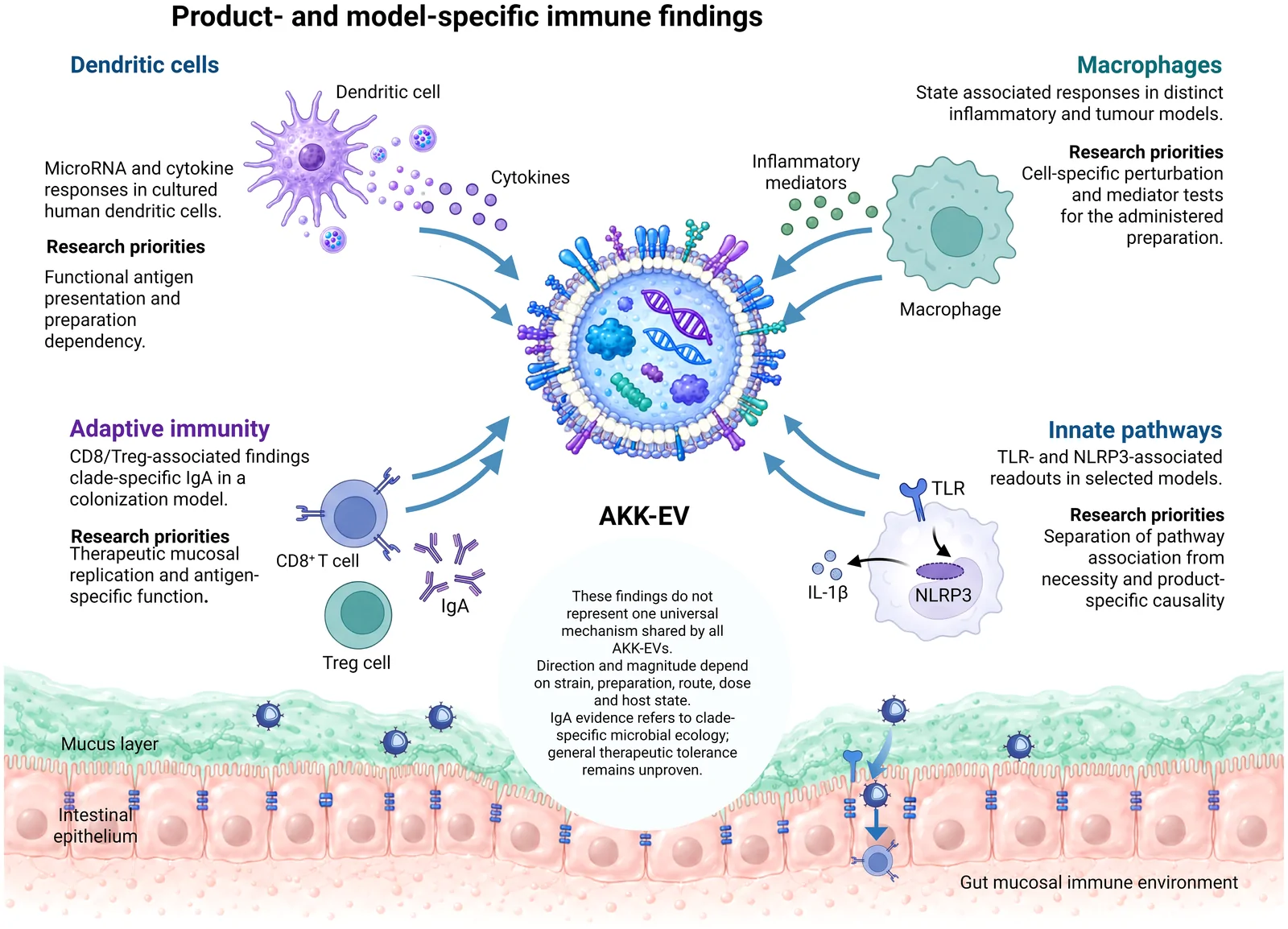
Immune responses to AKK-EV preparations. Four groups summarise reported observations and the experiments needed to determine their relevance to treatment. Dendritic-cell studies describe microRNA and cytokine responses, with antigen-presentation assays needed to define function. Macrophage studies report state-associated changes in inflammatory and tumour models, for which cell-specific perturbation and mediator testing are relevant. Adaptive-immune findings include CD8 T-cell and regulatory T-cell responses, together with clade-specific IgA observations from microbial colonisation. TLR- and NLRP3-associated readouts identify candidate innate pathways for causal testing. The central vesicle links these research areas schematically. The effects remain specific to strain, preparation, route, dose, and host state; clade-related IgA findings concern microbial ecology rather than an established general mechanism of therapeutic tolerance. Created in BioRender. Song, Y. (2026) https://BioRender.com/vr04sts.

### 4.3 Adaptive immune regulation in metabolic disease and cancer

The adaptive response may be useful in different ways across disease settings. Cytotoxic T-cell and macrophage changes accompanied tumour control in prostate-cancer models (47). A diabetes model showed partial dependence on a Treg-related pathway (48). In chronic-stress-associated colorectal tumourigenesis, oral OMVs reduced tumour burden, and Fetuin-A replacement attenuated the benefit in a related report (49, 50). These findings support a disease-specific treatment hypothesis. Work on Amuc_1434 concerns a bacterial component, with the published correction considered alongside the original report (51, 52). Selecting an immune-active EV product therefore requires evidence for the particular preparation, disease and route.

### 4.4 Intestinal and hepatic effects in metabolic liver disease

Oral AKK-EVs could be useful in metabolic liver disease if altering intestinal function produces a sustained hepatic benefit. High-fat-diet studies reported lower weight gain and changes in metabolic and inflammatory measures (53, 54). An earlier small EV experiment found reduced weight gain and cholesterol but no significant improvement in serum glucose, illustrating that metabolic outcomes need not move together (55). Related HFD/carbon tetrachloride experiments reported liver injury- and fibrosis-associated responses (56, 57), while a PEG-enriched preparation reduced hepatic lipid accumulation in a diet-induced metabolic dysfunction-associated steatohepatitis (MASH) model (58). Toxin-assisted injury and reduced liver fat address different stages of disease; neither alone establishes reversal of established MASH fibrosis.

Hepatic benefit could arise from altered permeability, portal exposure to microbial products, food intake, adipose inflammation, or metabolites. Transcriptional responses in HepG2 cells exposed to chemically extracted bacterial membrane material do not demonstrate treatment of liver disease (59). A gut-directed medicine needs a reproducible relation between intestinal action and hepatic improvement. A liver-delivery carrier additionally needs evidence that active cargo reaches the relevant hepatic cells.

Fibrosis and associated metabolic risk are central to the clinical assessment of MASLD (60). Histological MASH resolution without worsening fibrosis, or fibrosis improvement without worsening disease activity, sets a more demanding therapeutic benchmark, illustrated by the resmetirom trial (61). An AKK-EV programme should therefore test disease after establishment, measure intake and body weight, and use pair-fed controls when these change. Blinded histology and fibrosis assessment should be paired with a prespecified intestinal functional outcome. A hepatic effect that persists beyond weight change would give a gut-directed treatment a clearer clinical rationale.

### 4.5 Systemic and neurobehavioural effects of AKK-EVs

Extraintestinal experiments extend the possible indications but require product- and route-specific exposure and safety evidence. Reports describe blood-pressure- and preeclampsia-associated responses (62, 63), and sonication-derived particles in experiments involving postoperative cognitive dysfunction and pulmonary fibrosis (64, 65). In mastitis, vesicles were tested in cells and mice, whereas cows received whole bacteria (66). Disc degeneration and vascular calcification studies examined EV preparations alongside candidate cargo proteins (67, 68). Skeletal work likewise separates purified Amuc_1473 from oral or intravenous AKK-EVs that improved bone measures in ovariectomised mice (27, 69). In a sarcopenia report, the EV intervention was confined to C2C12 cells; the animal benefit followed treatment with live bacteria (70). These experiments identify different candidates and cannot be combined into a single systemic EV efficacy claim.

Neurobehavioural studies include oral EV treatment in several models (71–73). Serotonin-pathway responses in mice and Caco-2 assays support mechanistic investigation, but do not by themselves establish an antidepressant treatment or brain delivery (74, 75). Renal transcriptional findings after intravenous EV administration describe a pharmacodynamic response; that report reused tissues from the earlier hypertensive-rat experiment and is not an independent animal replication (62, 76). A species-comparison report remains source-limited (77).

Host physiology can alter the response to a microbial intervention. Greer et al. found that interferon-γ regulated AKK abundance and that restoring the bacterium improved glucose tolerance in their model (78). This whole-organism experiment shows how host immunity can influence a metabolic response. In EV development, diet, baseline inflammation, and the treatment stage should therefore be prespecified and measured where relevant. The mixed clinical and barrier findings above further argue for a defined population and functional endpoint.

## 5 Engineered EVs for therapeutic delivery

Engineering changes what must be demonstrated. A native preparation is usually tested for its combined biological activity; a loaded or modified product is intended to deliver a selected payload or alter its action. Its efficacy and safety depend on the full formulation and treatment regimen. Carrier choice should follow the delivery problem.

### 5.1 Composition and integrity after engineering

Loading and surface modification can alter membrane integrity, aggregation, surface proteins, and inflammatory activity. The final formulation should be characterised after loading, purification, and storage. Free payload and material released during processing also need measurement because they can contribute to efficacy or toxicity. Characterisation of the starting vesicles is insufficient to describe the administered construct.

### 5.2 Target selection and comparator design

Mammalian EVs, lipid nanoparticles, and other vesicle-based systems provide established concepts for evaluating cargo delivery (79, 80). The wider EV-therapy literature also describes the opportunities and constraints of vesicles as therapeutic tools (81). For an AKK-derived carrier, comparisons should hold payload, route, and target-engagement assay constant. Delivery to tumour cells, hepatocytes, or myeloid cells can produce different consequences within the same organ, so whole-organ fluorescence is an incomplete targeting endpoint.

### 5.3 Cargo-loaded vesicles in disease models

The HCC programme combined PD-L1 small interfering RNA (siRNA), a metal–organic framework, and an EV coating with ultrasound treatment (82). Tumour suppression in mouse and patient-derived models supports the complete construct. Development would require tumour-cell target engagement, exposure in nontumour liver, and tolerability in the setting of impaired hepatic reserve. Another design used targeted microRNA delivery for postmenopausal osteoporosis (83). Oral siRNA delivery and intravenous codelivery of Atox1 siRNA with elesclomol provide distinct tumour-treatment examples (84, 85). Empty carriers, matched free payload, and constructs lacking individual functional components would establish what the vesicle adds. For oral delivery, mucus penetration should also be assessed alongside effects on mucosal integrity.

### 5.4 Hybrid vesicles and combination therapy

Hybrid EV–lipid nanovesicles have been studied for plasmid delivery and cancer immunotherapy (86). A separate breast-cancer construct fused AKK OMVs with targeted Escherichia coli OMVs and loaded a copper chelator (87). Its activity is attributable to the combined product and dosing regimen. Component proportions, membrane integrity, payload retention, and potency should be measured in the final formulation. Matched free payload, each membrane component, and a nonhybridised mixture would help establish whether hybridisation improves treatment.

### 5.5 Therapeutic contribution of the EV carrier

Lipid-nanoparticle chemistry and RNA-delivery research offer useful principles for comparator design (88, 89). Head-to-head experiments should match administered payload, route, and schedule, then measure target engagement and toxicity alongside efficacy. A carrier could be useful through greater activity at a matched payload dose, lower toxicity at equivalent activity, or an effective route not available with the alternative. The comparison should specify which of these advantages is being tested.

Purified bacterial components remain relevant even in an engineering programme. Comparing an intact preparation with its active component can help determine whether membrane packaging is necessary. Manufacturing yield, storage, and dosing practicality should enter the decision together with biological activity; additional formulation complexity is justified when it produces a measurable treatment advantage.

## 6 Preclinical requirements for human studies

A candidate should advance only when the final formulation can be reproduced, produces a disease-relevant effect and has an acceptable safety profile for its intended route. Prespecified criteria allow an unfavourable result to guide reformulation or stop development. **Box 3** summarises the proposed decisions.

### 6.1 Batch consistency and release testing

Release testing should connect the strain bank and manufacturing record to particle integrity, impurities, microbial contamination and a mechanism-relevant functional assay. International Council for Harmonisation of Technical Requirements for Pharmaceuticals for Human Use (ICH) Q6B provides general specification principles that can inform this work (90). Particle count and protein content are complementary dose descriptors; neither is a substitute for potency. Acceptance ranges must be justified for the particular AKK-EV product.

Genome and passage records should remain linked to each production lot. Changes in strain, medium, harvest, or purification should be compared with a reference preparation. Shared reference material and harmonised assays can help separate variation in manufacture from variation in measurement.

### 6.2 Potency assays and disease-relevant activity

The potency assay should measure an activity relevant to the proposed treatment. For a mucosal product, this could be the recovery of barrier function or mucus function under defined injury conditions. A metabolic product needs an assay linked to its in vivo effect; a loaded carrier needs cargo-specific target engagement. Cytokine or protein measurements are useful when their relation to functional benefit has been established.

Process blanks, EV-depleted fractions and membrane disruption help distinguish activity of vesicular and nonvesicular material. A named mediator requires a separate necessity test, with rescue where feasible. The results can also identify a simpler fraction or purified component worth developing.

### 6.3 Dose selection and target-site exposure

Dose reporting should specify formulation, particle and protein measurements, route, frequency, and duration. For oral treatment, gastric and intestinal stability, local retention, and degradation may be more informative than plasma pharmacokinetics. Injected formulations require target- and nontarget-tissue exposure and clearance measurements after repeated dosing. Escalation studies should assess the intended activity together with inflammatory responses.

The exposure assay should follow the therapeutic mechanism. Local intestinal activity followed by hepatic improvement is compatible with an indirect gut–liver effect. A direct-delivery claim requires recovery of the relevant particle-associated material or evidence that active cargo reaches its cellular target. Fluorescence alone leaves these possibilities unresolved.

### 6.4 Route-specific safety and patient selection

Safety studies should use the final product and a clinically relevant dosing schedule. Oral preparations require assessment of epithelial injury, local inflammation, and repeated exposure. Injected and engineered products also require product-appropriate testing of cytokine responses, complement and coagulation effects, organ injury, and repeat-dose tolerance. Active ulceration, advanced liver disease, and immunosuppression deserve attention because they can alter exposure or susceptibility.

The food-safety assessment of pasteurised A. muciniphila applies to the assessed preparation and use (91). Concentrated EV fractions and injected constructs need their own evidence. Benefit and toxicity should be evaluated at exposures achievable with reproducible lots, including a justified margin around the intended dose.

### 6.5 Storage stability and regulatory planning

Stability testing should include retained potency, impurities, and integrity under defined storage conditions. ICH Q1A(R2) offers general stability principles (92). Together with the cell-bank, comparability, and specification concepts above, these can inform development without supplying an AKK-EV-specific regulatory pathway. Material used for confirmatory safety testing should represent the intended clinical formulation.

The composition, intended claim, route, and jurisdiction will determine regulatory classification. Early consultation should address product definition, manufacturing controls, potency, and nonclinical testing. This is particularly important when a food-associated organism is used to produce a concentrated fraction or a multicomponent therapeutic construct.

## 7 Discussion

### 7.1 Therapeutic value in mucosal and hepatic disease

The translational case for AKK-EVs rests on delivering a useful microbial activity in a form that can be manufactured and dosed consistently. Local mucosal treatment offers a practical setting in which to test this proposition. The intended site is accessible after oral administration, and functional recovery can be assessed alongside inflammation and tissue injury. Limited systemic exposure could be compatible with such a treatment. The decisive comparison is whether an EV preparation improves recovery after disease onset relative to pasteurised bacteria or the relevant active component. This would establish what purification into a vesicle fraction adds to treatment.

For liver disease, therapeutic benefit and hepatic delivery are separate questions. An oral preparation could improve liver injury through intestinal or metabolic effects without intact vesicles entering hepatocytes. Paired intestinal and hepatic measurements would help determine whether these effects accompany one another. Pair-feeding and assessment of body weight are needed to separate a hepatic response from changes in energy intake or weight gain. Treatment of established disease, with histological and fibrosis outcomes, would provide a stronger basis for development than prevention alone. The choice between a gut-directed treatment and a liver-targeted carrier should follow from these experiments.

Engineered AKK-EV products require a different comparison because the payload, vesicle membrane, and other carrier components may each contribute to efficacy. Matching payload exposure while removing or replacing the EV component would test whether the membrane improves delivery or therapeutic index. For liver tumours, that assessment should distinguish tumour from nontumour liver exposure and relate target engagement to tissue injury. A favourable result would support a specific carrier–payload combination, rather than a general claim about native AKK-EVs. If a simpler formulation gives comparable benefit and tolerability, the additional manufacturing demands of the EV construct need a clear justification.

These comparisons should guide candidate selection before a programme proceeds towards human studies. Whole-cell human trials provide a clinical basis for comparison, but the efficacy and safety evidence for isolated AKK-EVs remains preclinical. A candidate should show reproducible functional benefit, acceptable repeat-dose safety for its intended route, and an advantage over a justified active comparator. Manufacturing and biological testing need to use the same defined formulation so that a positive result can inform subsequent development. **Box 3** brings these decisions together; its three-lot planning minimum is an author proposal that requires external evaluation. Oral mucosal treatment is a testable starting point, not an established clinical priority.

### 7.2 Evidence gaps and methodological limitations

The main uncertainty is whether reported effects persist when preparation and experimental setting change. Studies differ in strain, culture, purification, dose units, treatment timing, and controls. Some reports also contain discrepancies between methods and figure legends, including treatment duration or dose; these are retained in **Supplementary Table S3**. Prevention and concurrent-treatment designs leave the response in established disease less well defined. Domain-level appraisal identifies frequent uncertainty in allocation, masking, and analysis, which cannot be resolved by a positive biological result. Counting related reports separately broadens the publication list without necessarily providing independent replication. Neither the 50-report count nor the 46-group sensitivity count establishes clinical priority.

The synthesis is limited by variation in vesicle preparation, incomplete experimental reporting, and uncertain independence among some related reports. Cell-only and abstract-level evidence provides a narrower basis for interpretation than controlled animal intervention studies. The four-question framework was developed for this review; its reliability and predictive value have not been tested in independent studies. Further controlled comparisons could change the relative promise of oral mucosal, systemic, and engineered applications. SANRA informs appraisal of the narrative review (93).

## 8 Conclusion

AKK-EVs merit further investigation as local mucosal treatments, mediators of gut–liver effects, and components of engineered delivery systems. Their clinical value will depend on the advantage of a reproducible vesicle preparation over an appropriate whole-cell product, active component, or carrier. The strongest next steps are functional confirmation after disease onset, testing of the relevant site of action, and direct comparison at justified exposure levels.

Human whole-cell trials provide useful clinical comparators, while isolated AKK-EV efficacy and safety remain preclinical. Progression requires consistent batches, route-specific safety, and a disease-relevant benefit that supports the selected product.
