# Supplementary material for: From mucosal activity to medicinal products: translational evidence for Akkermansia muciniphila extracellular vesicles
Source: Front Immunol. 2026 Oct 26;17:1942618. doi: 10.3389/fimmu.2026.1942618 (PMC13746187; doi:10.3389/fimmu.2026.1942618)
Supplement: Supplementary file 1 [file Table1.docx]

Supplementary material

Five tables describe the literature sources and evidence from 50 publications available by 19 July 2026. Tables S2–S4 link findings, preparations and experimental controls. Table S5 reports domain-level animal-study appraisal. Stable study identifiers connect all four evidence tables.

Abbreviations: EV, extracellular vesicle; OMV, outer membrane vesicle; PK, pharmacokinetics; BD, biodistribution; NR, not reported in the retained source. In Table S5, NA means not applicable to the assessed animal EV intervention; it is not a risk rating.

# Supplementary Table S1 Search strategies and study selection

Core PubMed/MEDLINE search: 22 July 2026; publication eligibility cutoff: 19 July 2026. The strategy combines bacterial-source and vesicle concepts with AND, and synonyms with OR, using title/abstract tags [tiab]. Additional disease and delivery queries overlap the core search. The execution date of the retained registry query is unverified.

| **Source and purpose** | **Recorded search or retrieval method** | **Recorded yield and use** |
| --- | --- | --- |
| PubMed/MEDLINE core search | ("*Akkermansia muciniphila*"[tiab] OR *Akkermansia*[tiab]) AND ("extracellular vesicle"[tiab] OR "extracellular vesicles"[tiab] OR "outer membrane vesicle"[tiab] OR "outer membrane vesicles"[tiab] OR OMV[tiab] OR OMVs[tiab] OR "membrane vesicle"[tiab] OR "membrane vesicles"[tiab]) | 107. Recovered core query and 107 PubMed identifiers. |
| PubMed/MEDLINE engineering and delivery | ("*Akkermansia muciniphila*"[tiab] OR *Akkermansia*[tiab]) AND ("extracellular vesicle"[tiab] OR "outer membrane vesicle"[tiab] OR OMV[tiab] OR vesicles[tiab]) AND (engineering[tiab] OR engineered[tiab] OR delivery[tiab] OR siRNA[tiab] OR miRNA[tiab] OR nanocarrier[tiab] OR biodistribution[tiab]) | 17 identifiers; overlapping, not additive. Engineering and exposure update. |
| PubMed/MEDLINE disease-focused search | ("*Akkermansia muciniphila*"[tiab] OR *Akkermansia*[tiab]) AND ("extracellular vesicle"[tiab] OR "outer membrane vesicle"[tiab] OR OMV[tiab] OR vesicles[tiab]) AND (barrier[tiab] OR colitis[tiab] OR inflammation[tiab] OR obesity[tiab] OR liver[tiab] OR cancer[tiab] OR brain[tiab] OR cognitive[tiab] OR asthma[tiab] OR fibrosis[tiab] OR hypertension[tiab] OR pregnancy[tiab] OR diabetes[tiab]) | 91 identifiers; overlapping, not additive. Disease and distant-organ update. |
| ClinicalTrials.gov | *Akkermansia* AND (extracellular vesicle OR extracellular vesicles OR outer membrane vesicle OR outer membrane vesicles OR OMV OR OMVs) | Targeted check for isolated *AKK*-EV interventions; no exhaustive multi-registry search is claimed. |
| Publisher pages and citation tracking | Reference lists from primary reports and reviews; title and identifier searches. | Additional reports retained: Raftar et al. (ST06, disease-focused query), Wang et al. (ST24, SciOpen full text) and Xu et al. (ST42, retained full text). |

Eligibility: original cell, animal or human observational studies examining *AKK* vesicle preparations or EV-associated components were included. Reviews, editorials, records without original data, unrelated studies and post-cutoff publications were excluded from the study-level synthesis. Whole-cell-only interventions, other bacterial EVs and synthetic carriers informed background or comparator choice. Duplicate versions and corrections were considered with the parent report. Final publications were preferred; retained abstracts, previews and linked preprints are labelled in the evidence tables. Section 2.1 gives the full criteria.

Selection: two reviewers independently screened and assessed the records and agreed on the final decisions. The core list contains 47 included records, 59 exclusions and one unresolved record. Three additional-source reports bring the total to 50: Raftar et al. (ST06; 14 February 2022), Wang et al. (ST24; 28 February 2026) and Xu et al. (ST42; online 14 April 2026). A fourth candidate, Zou et al., appeared on 22 July 2026 and was excluded after the cutoff. ST36, ST45, ST47, ST49 and ST52 were excluded following the two-reviewer eligibility reassessment dated 29 July 2026. Their recorded reason is that they did not meet the requirements of the review; criterion-specific details remain to be supplied. The workbook preserves the source reconciliation and decision history.

# Supplementary Table S2 Study models, findings and therapeutic relevance

Stable identifiers ST01–ST55 are retained for traceability; ST36, ST45, ST47, ST49 and ST52 have been excluded, leaving 50 publications in Tables S2–S5. Reference numbers refer to the main bibliography. Related pairs are ST03/ST04 (F03), ST05/ST06 (F05), ST11/ST37 (F12) and ST17/ST30 (F16). ST37 explicitly reused tissues from ST11; the other three links remain provisional. Collapsing these four pairs gives 46 provisional groups, not 46 confirmed independent experiments. ST35 is retained as a single report after exclusion of ST36. Human measurements and purified-protein interventions are labelled separately from intact-EV treatment.

| **Study and source** | **Experimental model** | **Preparation tested** | **Main finding** | **Therapeutic interpretation and limitations** |
| --- | --- | --- | --- | --- |
| ST01 / F01 Chelakkot et al., 2018 Reference 31 DOI 10.1038/emm.2017.282 | HFD-fed C57BL/6 male mice; Caco-2 epithelial monolayers; human fecal EV metagenomic comparison | Native *A. muciniphila*-derived EVs | Reduced HFD-associated gut permeability, improved tight-junction markers, and activated AMPK-dependent barrier signaling | EV cargo causality and product-purity controls are limited; human data are compositional/contextual rather than interventional |
| ST02 / F02 Ashrafian et al., 2019 Reference 32 PMID 31191842 | Human Caco-2 intestinal epithelial/colorectal adenocarcinoma cell-line model treated for 24 h with live *A. muciniphila* or *A. muciniphila* EVs; qPCR readouts for TLR and tight-junction genes | Native *A. muciniphila* MucT/ATCC BAA-835 extracellular vesicles; live *A. muciniphila* comparator | Live *A. muciniphila* increased TLR2/TLR4 and multiple tight-junction gene transcripts; *A. muciniphila* EVs decreased TLR2/TLR4 at selected doses and increased selected tight-junction transcripts, especially OCLN and ZO2; no viability difference was detected after 24 h | Single-cell-line, short-exposure transcriptional study; EV dose units are reported inconsistently as ug and ug/mL; no animal efficacy, permeability functional assay, cargo identification, receptor perturbation, EV-depleted/heat/protease controls, biodistribution or PK; SEM rather than TEM/NTA was used for EV characterization |
| ST03 / F03 Ashrafian et al., 2019 Reference 53 DOI 10.3389/fmicb.2019.02155 | Male C57BL/6 diet-induced obesity model: 3 months HFD induction followed by 5-week treatment while continuing HFD; normal-diet comparison arms; Caco-2 monolayer assays for epithelial/inflammatory gene and cytokine responses | Native *A. muciniphila* MucT/ATCC BAA-835 extracellular vesicles; live *A. muciniphila* comparator | Oral *A. muciniphila* EVs reduced HFD-associated body-weight gain, food intake, epididymal adipose weight/adipocyte size, plasma total cholesterol and glucose, improved colon mucosal thickness/crypt depth and upregulated tight-junction transcripts while lowering selected inflammatory/TLR4 signals; Caco-2 experiments supported direct epithelial effects | Small animal groups and exclusions after HFD induction; EV frequency not explicit in XML; no EV-depleted supernatant, heat-inactivated EV, cargo-depletion, receptor blockade or rescue controls; mostly transcriptional barrier readouts without direct permeability assay details in extracted XML; no formal PK/biodistribution/toxicology |
| ST04 / F03 Ashrafian et al., 2021 Reference 54 DOI 10.1186/s12934-021-01709-w | Prevention study in 8-week-old male C57BL/6 mice fed HFD for 5 weeks with live *A. muciniphila*, pasteurized *A. muciniphila* or EV treatment; liver, colon, eWAT, plasma cytokine/metabolic and stool microbiota qPCR readouts | Native *A. muciniphila* MucT/ATCC BAA-835 extracellular vesicles; live and pasteurized *A. muciniphila* comparators | EVs and pasteurized/live *A. muciniphila* prevented HFD-associated weight gain, food intake and metabolic/cytokine changes; EVs improved liver steatosis/inflammation markers, colon tight-junction/inflammatory gene expression, eWAT adipocyte size/inflammation and selected gut dysbiosis indices | Prevention rather than reversal design; route/frequency not explicit in XML despite 200 uL dosing; EV characterization limited mainly to SEM/SDS-PAGE in extracted text; no EV-depleted, heat-inactivated EV, cargo-depletion, receptor blockade or rescue controls; no formal PK/biodistribution/toxicology |
| ST05 / F05 Raftar et al., 2021 Reference 56 DOI 10.1128/spectrum.00484-21 | HFD/CCl4-induced liver injury/fibrosis model in male C57BL/6 mice with daily 4-week intervention; LPS-activated LX-2 human hepatic stellate cell model; liver, colon, eWAT, kidney and targeted stool microbiota readouts | Native *A. muciniphila* MucT/ATCC BAA-835 EVs; live *A. muciniphila* and pasteurized *A. muciniphila* comparators | *A. muciniphila* EVs downregulated TLR2/TLR4 and fibrosis markers in LPS-activated LX-2 cells; daily oral EVs improved HFD/CCl4 liver and colon histopathology, serum cytokines, ALT/AST, glucose/lipid indices, hepatic fibrosis/inflammation markers, colon barrier/inflammatory markers and selected adipose/kidney fibrosis/inflammation readouts | Small n=5 animal groups; HFD/CCl4 injury model may not generalize to chronic human liver disease; no EV-depleted supernatant, heat-inactivated EV, cargo-depletion, receptor blockade or rescue controls; no formal EV biodistribution, PK, immunogenicity or GLP-style toxicology |
| ST06 / F05 Raftar et al., 2022 Reference 57 DOI 10.1038/s41598-022-06414-1 | HFD/CCl4-induced liver injury model in male C57BL/6 mice with daily 4-week intervention; LPS-activated LX-2 hepatic stellate cells; colon histometry/barrier genes, liver inflammation/PPAR genes and white-adipose inflammatory readouts | Native *A. muciniphila* MucT/ATCC BAA-835 EVs; live *A. muciniphila* and pasteurized *A. muciniphila* comparators | EVs, live and pasteurized *A. muciniphila* reduced TLR5/TLR9 inflammatory signaling and increased PPAR-alpha/gamma in activated LX-2 cells and HFD/CCl4 mouse tissues; EV treatment strongly improved colon histology/tight-junction markers, liver histology/cytokines/inflammatory genes and adipose IL-6/TNF-alpha/IL-10 and PPAR readouts | Small n=5 animal groups; closely related to prior HFD/CCl4 *A. muciniphila* derivative work and should be interpreted as complementary inflammatory-axis evidence; no EV-depleted, heat-inactivated EV, cargo-depletion, receptor blockade, biodistribution, PK or formal toxicology package |
| ST07 / F04 Luo et al., 2021 Reference 47 DOI 10.2147/ijn.s304515 | Immune-competent C57BL/6 male mice bearing subcutaneous RM-1 syngeneic prostate cancer; RM-1, DU145 and PC-3 prostate cancer cells; THP-1 and RAW264.7 macrophage assays; RAW264.7, BPH-1, HMEC and VSMC normal-cell tolerance panel | Native *A. muciniphila*-derived extracellular vesicles (*Akk*-EVs) | Intravenous *Akk*-EVs reduced RM-1 tumor burden, increased GZMB+ and IFN-gamma+ CD8+ T cells, recruited macrophages and shifted them toward an M1-like phenotype; conditioned medium from *Akk*-EV-treated macrophages suppressed prostate cancer cell proliferation and invasion | Single subcutaneous syngeneic PCa model; no tumor biodistribution/PK; no EV-free fraction, soluble-cargo, cargo-loss or immune-cell depletion/rescue controls; mechanism remains immune-associative rather than fully causal |
| ST08 / F06 Chen et al., 2023 Reference 63 DOI 10.1002/jev2.12328 | L-NAME-induced preeclampsia model in pregnant C57BL/6J mice; live *A. muciniphila* pretreatment arm; oral AmEV intervention arm; PKH26-AmEV biodistribution; HTR-8/SVneo trophoblast uptake and hypoxia/reoxygenation model; placental transcriptome and EGFR-PI3K-AKT readouts | Native *A. muciniphila* ATCC BAA-835 EVs (AmEVs); live *A. muciniphila* comparator; PBS vehicle controls | AmEVs showed a mean NTA diameter of 119.2 +/- 3.4 nm, spherical bilayer morphology and OmpA/LPS signal; fluorescent signal from labelled AmEV preparations was detected in placenta and foetuses within 5 h, with uptake-associated signal in HTR-8/SVneo cells; intact-vesicle delivery was not established; every-other-day oral AmEVs lowered L-NAME-associated SBP and urine protein, improved foetal mass and placental efficiency, reduced placental lesions and supported spiral-artery/trophoblast remodelling; AmEVs activated placental and trophoblast EGFR-PI3K-AKT signalling and improved trophoblast proliferation/migration/invasion in vitro | Mainly preventive/preconception dosing in an L-NAME mouse model; dye-based biodistribution is short-term and not quantitative PK; no EV-depleted supernatant, heat-inactivated EV, soluble/free-cargo, cargo-depletion, EGFR/PI3K blockade/rescue or strain/clade comparator; no formal immunogenicity or GLP repeat-dose toxicology package; human relevance limited to HTR-8/SVneo cells and prior clinical-microbiome association |
| ST09 / F07 Zheng et al., 2023 Reference 33 DOI 10.3390/nu15224722 | DSS-induced acute colitis prevention model in male C57BL/6J mice; RAW264.7 macrophage uptake and LPS-inflammatory model; colon histology, mucus/goblet cell, serum permeability/inflammatory markers and gut microbiome sequencing | Native *A. muciniphila* ATCC BAA-835 EVs; live *A. muciniphila* comparator | *Akk* EVs were internalized by RAW264.7 cells and suppressed LPS-induced NO, IL-1beta and TNF-alpha; daily oral *Akk* EVs reduced DSS-associated weight loss, DAI score, colon shortening, histological injury, mucus loss, inflammatory cytokines, DAO/D-LA permeability markers and restored MUC2/ZO-1 and microbiota structure | Primarily prevention rather than rescue design; no EV-depleted supernatant, heat-inactivated EV, soluble/free-cargo, cargo-depletion, receptor blockade or rescue controls; no formal biodistribution, PK, immunogenicity or repeat-dose toxicology package; live comparator included but no strain/clade comparator |
| ST10 / F08 Mofrad et al., 2024 Reference 41 DOI 10.1007/s12602-023-10058-6 | Human monocyte-derived dendritic cells generated from healthy-donor peripheral blood mononuclear cells. | *A. muciniphila* MucT OMVs, compared with live bacteria, LPS, dexamethasone and PBS. | OMV and bacterial treatments altered dendritic-cell microRNAs, surface markers and IL-10/IL-12 secretion. | In vitro only. Vesicle dependency, cargo attribution and clinical tolerability were not tested. The text reports experiments performed twice, whereas the statistics section states at least three independent biological experiments, creating a replication-reporting inconsistency. |
| ST11 / F12 Kim et al., 2024 Reference 62 DOI 10.1038/s41440-024-01627-5 | Male spontaneously hypertensive and Wistar-Kyoto rats; ex vivo Sprague-Dawley aortic rings and splenic T-cell assays. | Native *A. muciniphila* EV preparation administered intravenously. | Weekly injections attenuated systolic blood-pressure rise in hypertensive rats and altered immune readouts, whereas the preparation did not directly relax isolated aortic rings. | No blinded outcome assessment, sample-size justification, biodistribution or immune-cell necessity experiment. Absence of observed body-weight toxicity is not a formal systemic safety package. |
| ST12 / F09 Gao et al., 2024 Reference 64 DOI 10.1016/j.bbamcr.2023.119630 | Male C57BL/6J mice with intestinal ischaemia-reperfusion-induced postoperative cognitive dysfunction; mouse microglial cells. | Particles generated by sonication of harvested *A. muciniphila* cells and ultracentrifugation; not a native secreted-EV preparation. | Oral treatment improved behavioural, intestinal/brain barrier and inflammatory readouts. PLX5622-mediated microglial ablation attenuated selected effects. | The administered particles were created by bacterial sonication and should not be pooled with native secreted *AKK*-EVs. Protein- and particle-based dose statements conflict. PLX5622 tests microglial contribution, not TLR2/4 necessity; no exposure was measured. |
| ST13 / F10 Raftar et al., 2024 Reference 65 DOI 10.1016/j.biopha.2024.117571 | Male C57BL/6 mice receiving high-fat diet with or without carbon tetrachloride; lung gene-expression and cytokine endpoints. | *A. muciniphila* EV-enriched preparation compared with live and pasteurized bacteria. | Treatments altered lung inflammatory/fibrotic transcripts and IL-6/IL-10 in the HFD/CCl4 setting. | EV filtration, morphology, size and purity were not reported. Pulmonary fibrosis was not confirmed by lung histopathology or protein-level fibrosis measures. Route/frequency were incompletely reported, and no exposure or mechanistic perturbation was performed. |
| ST14 / F11 Zhao et al., 2025 Reference 36 DOI 10.3390/microorganisms13020464 | In vitro *AKK*-EV characterization and anti-inflammatory testing in LPS-stimulated Caco-2 intestinal epithelial cells; EV proteomics, transcriptome analysis and MAPK-pathway western blot validation | Native *A. muciniphila* MucT/ATCC BAA-835-derived EVs (Am-EVs); no live-bacterium comparator reported | Am-EVs were 50-200 nm double-membrane vesicles with mean particle diameter 92.48 +/- 0.28 nm and protein concentration 14.50 +/- 2.23 mg/mL; HPLC-MS identified 850 proteins; Am-EVs increased Caco-2 viability under LPS challenge, reduced NO, ROS, MDA and TNF-alpha/IL-1beta/IL-6 mRNA in a dose-responsive pattern, increased CAT activity and modulated MAPK-related transcriptomic/protein signals including lower TRIF, MyD88, p38 MAPK and FOS and higher TGFBR2 | No animal disease model, oral delivery, biodistribution, PK, immunogenicity or toxicology; no live-bacterium, EV-depleted supernatant, heat-inactivated EV, soluble/free-cargo, cargo-depletion, receptor blockade or rescue controls; 0.45 um prefiltration is less stringent than 0.22 um and may leave larger particles; pathway causality inferred from RNA-seq/WB rather than perturbation experiments |
| ST15 / F13 Noori et al., 2025 Reference 42 DOI 10.1159/000543446 | STC-1 mouse intestinal neuroendocrine tumour-cell line. | *A. muciniphila* EV-enriched preparation compared with viable *A. muciniphila* and PBS. | EV exposure altered enteroendocrine, endocannabinoid, Toll-like-receptor and cytokine readouts. | In vitro tumour-derived cell line only. No vesicle-dependency, cargo perturbation or functional barrier endpoint. The methods state bacterial MOI 10, while results describe MOI 50 and 100. |
| ST16 / F14 Zhang et al., 2025 Reference 66 DOI 10.1096/fj.202500877rr | Bovine subclinical mastitis milk-microbiota association study; MAC-T bovine mammary epithelial LPS-inflammation model; GW4869 suppression of AOMV secretion from live *A. muciniphila*; isolated AOMV uptake and anti-inflammatory testing; DiD-AOMV mouse mammary tissue distribution; LPS-induced mouse mastitis model; preliminary dairy-cow live *A. muciniphila* supplementation | Native *A. muciniphila*-derived outer membrane vesicles (AOMVs); live *A. muciniphila* and pasteurized *A. muciniphila* comparators; GW4869-treated *A. muciniphila* secretion-inhibition condition | Mastitis milk showed reduced *Akkermansia*/Verrucomicrobia signals linked to inflammatory markers; live/pasteurized *A. muciniphila* lowered LPS-induced inflammatory cytokines and TLR4/NF-kappaB activation in MAC-T cells; GW4869 reduced AOMV protein/particle output and attenuated live-*Akk* anti-inflammatory activity; isolated AOMVs showed spherical membrane morphology, were internalized by MAC-T cells, reduced TNF-alpha, IL-6 and IL-1beta and inhibited TLR4/I-kappaB/p65 signalling; DiD-AOMVs accumulated in mouse mammary gland after tail-vein injection and alleviated LPS-induced mammary histological injury and TNF-alpha/IL-1beta; live-*Akk* supplementation improved SCC/CMT readouts in a small dairy-cow subclinical mastitis cohort | AOMV efficacy used tail-vein delivery rather than oral delivery; cow intervention used live *A. muciniphila*, not purified AOMVs; small animal and cow sample sizes; GW4869 is an indirect secretion inhibitor and may have off-target interpretation limits; no EV-depleted supernatant, heat-inactivated AOMV, soluble/free-cargo, cargo-depletion, receptor blockade/rescue or quantitative PK/immunogenicity/GLP toxicology package |
| ST17 / F16 Jin et al., 2025 Reference 49 DOI 10.1080/19490976.2025.2555618 | Human CRC stress-association/tumor-tissue *Akkermansia* analysis; AOM/DSS colitis-associated colorectal cancer model combined with chronic unpredictable mild stress in male C57BL/6J mice; live *A. muciniphila* rescue; oral *A. muciniphila* OMV intervention; HCT116/SW620 CRC cell uptake, viability and colony-formation assays; OMV stability and proteomic profiling | Native *A. muciniphila* ATCC BAA-835 OMVs; live *A. muciniphila* comparator; *A. muciniphila* culture supernatant exploratory comparator | Chronic stress was associated with larger CRC/CAC tumor burden and reduced *A. muciniphila* abundance in mouse feces, mouse tumor tissues and human CRC tumor tissues; live *A. muciniphila* gavage increased fecal/tumor *Akk* abundance and reduced stress-associated body-weight loss, DAI, tumor number/load, Ki67 positivity and histopathological progression; purified OMVs averaged 110.60 +/- 60.3 nm, showed cup-shaped/spherical bilayer morphology, remained stable in simulated gastric/intestinal fluids and, after oral gavage, reduced tumor number/load, Ki67 positivity and histopathological scores in stressed CAC mice; Dil-labelled OMVs were taken up by HCT116 and SW620 CRC cells and reduced CRC cell viability/colony formation while sparing NCM460 cells; LC-MS/MS identified 226 proteins stably enriched in OMVs | OMV mechanism remains largely correlative/proteomic; no EV-depleted supernatant, heat-inactivated OMV, soluble/free-cargo, cargo-depletion, candidate-protein loss/rescue, receptor blockade or immune-cell depletion; 0.45 um filtration is less stringent than 0.22 um; no quantitative biodistribution/PK, immunogenicity or formal repeat-dose toxicology; clinical analysis limited by incomplete confounder adjustment and association rather than intervention |
| ST18 / F15 Hong et al., 2025 Reference 22 DOI 10.1038/s41467-025-57631-x | Human *A. muciniphila* clade-distribution datasets; in vitro AmI/AmII co-culture and growth-inhibition assays; germ-free C57BL/6, ASF gnotobiotic and JH-/- B-cell-deficient mouse colonization models | Clade-specific *A. muciniphila* EVs, especially EB-AMDK39/AmII-derived EVs and luminal/fecal EV-enriched fractions from AmII-associated germ-free mice | AmII-derived EVs inhibited AmI growth in vitro and from host luminal fractions; AmII strains outcompeted AmI during gut colonization; oral AmII-EV pretreatment induced clade-specific IgA and improved early AmII colonization in WT but not JH-/- mice | Not a disease-treatment or safety/PK study; EV cargo candidates require genetic/cargo-loss confirmation; findings show that *A. muciniphila* EV products are clade/strain-specific and should not be treated as interchangeable |
| ST19 / F17 Chen et al., 2026 Reference 72 DOI 10.1080/19490976.2025.2611546 | DSS-induced colitis-related cognitive impairment in 6-week-old male C57BL/6J mice; HT-22 hippocampal neuronal cells challenged with LPS; gut microbiota, SCFA, plasma metabolomics, hippocampal cytokine/5-HT and proteomics analyses | Native *A. muciniphila*-derived EVs (AmEVs) | Oral AmEVs attenuated DSS colitis, improved Y-maze/NOR cognitive readouts, reduced hippocampal neuroinflammation, restored barrier/glial/synaptic markers, remodeled microbiota/SCFAs, and rebalanced tryptophan/5-HT signaling with hippocampal AmEV uptake | Causal chain remains incomplete: microbiota shifts, Amuc 1100/TLR2, Tph2/5-HT and neural-route contributions were not genetically validated in vivo; TLR2 inhibition only partially reduced AmEV effects; no EV-depleted/free-cargo/cargo-loss controls evident |
| ST20 / F18 Chang et al., 2026 Reference 48 DOI 10.3389/fimmu.2026.1739048 | STZ-induced T1DM in male C57BL/6 mice; fecal *A. muciniphila* qPCR; pancreatic/hepatic histology; metabolic tolerance testing; oxidative-stress/inflammatory cytokine assays; pancreatic lymph-node and tissue Treg analyses; anti-CD25 Treg-depletion experiment | Native *A. muciniphila*-derived extracellular vesicles (AmEVs) | Daily oral AmEVs improved glucose homeostasis, insulin sensitivity, pyruvate tolerance, beta-cell/islet readouts, hepatic lipid/liver markers, oxidative stress and inflammatory cytokines; AmEVs expanded pancreatic Tregs and anti-CD25 depletion attenuated metabolic and anti-inflammatory benefits | STZ model reflects beta-cell injury with inflammatory/immune perturbation rather than spontaneous autoimmune diabetes; active EV cargo and receptor pathway not defined; no EV-depleted/free-cargo/cargo-loss controls; long-term safety and PK absent |
| ST21 / F19 Huang et al., 2026 Reference 82 DOI 10.1016/j.xcrm.2025.102524 | Hepa 1-6, H22 and Huh-7 HCC cells; female C57BL/6 orthotopic H22 HCC model; humanized NOG-EXL HCC-PDX model; patient-derived organoids (PDOs, n=3). | SMB, a core-shell siRNA-MOF@*A. muciniphila* BMV nanoformulation carrying PD-L1-targeting siRNA and a zinc/porphyrin sonodynamic MOF, activated with ultrasound. | The platform showed HCC accumulation, PD-L1 silencing, ultrasound-associated pyroptosis through the NLRP3/Caspase-1/GSDMD axis, TIME remodeling and trained-immunity-like macrophage activation, with antitumor activity in syngeneic and PDX/PDO HCC models. | Multi-component intervention: *A. muciniphila* vesicle targeting, MOF/ultrasound pyroptosis, PD-L1 siRNA and immune-training effects are not fully separable; macrophage-training epigenetics and broader HCC-subtype validation remain unresolved. |
| ST22 / F20 Ji et al., 2026 Reference 83 DOI 10.1186/s12951-026-04513-z | BMSCs and BMMs for osteogenic/osteoclast assays; female C57BL/6 ovariectomy model for postmenopausal osteoporosis and bone-targeting assessment. | Native *AKK*-EVs and engineered bone-targeted *AKK*-EVs (BT-*AKK*-EVs) modified with the SDSSD bone-targeting peptide. | SDSSD-engineered BT-*AKK*-EVs accumulated preferentially in bone after systemic injection, delivered/enriched miR-21-5p activity, promoted osteogenesis, inhibited osteoclastogenesis and alleviated OVX-associated bone loss through PI3K-AKT-linked signaling. | Engineering and IV delivery are early preclinical; EV purity, long-term immunotoxicity, dose scaling and source-data-level effect sizes require author/PDF/supplement verification. |
| ST23 / F21 Kang et al., 2013 Reference 34 DOI 10.1371/journal.pone.0076520 | DSS-induced acute colitis in C57BL/6 mice; CT26 epithelial cells and Raw264.7 macrophages | Native *A. muciniphila*-derived EVs from culture medium | *A. muciniphila* EVs decreased in DSS colitis-associated fecal EV profiles and orally administered AmEV ameliorated DSS-colitis phenotypes | Early EV-preparation standards; unit inconsistency for AmEV dose; no cargo perturbation or modern orthogonal purity controls |
| ST24 / F22 Wang et al., 2026 Reference 45 DOI 10.26599/nr.2026.94908421 | Male BALB/c mice with DNFB-induced atopic dermatitis; keratinocyte and reporter assays for miR-21-5p/TLR4. | Native *A. muciniphila* EV preparation administered orally or subcutaneously. | Treatment improved dermatitis and inflammatory readouts. miR-21-5p mimic/inhibitor and reporter experiments implicated TLR4, but the administered preparation was not depleted of miR-21-5p. | One model, n=6/group. Lipophilic-dye tracking did not establish intact-particle exposure. Safety sampling was limited and no batch-qualified cargo-loss/rescue experiment was performed. |
| ST25 / F23 Zhu et al., 2025 Reference 43 DOI 10.1016/j.intimp.2025.114195 | Male C57BL/6J mice exposed to cigarette smoke for 8 weeks; BPH-1 and RAW264.7 cell models. | Native *AKK*-EV preparation; live bacteria and recombinant P9 were separate comparators/interventions. | Oral *AKK*-EVs and recombinant P9 reduced prostate inflammatory and fibrotic readouts. IV-labelled EVs localized to prostate tissue in a separate tracing experiment. | Therapeutic dosing was oral but distribution tracing was intravenous. P9 sufficiency does not establish its necessity within the EV preparation. Randomization, blinding and sample-size justification were not reported. |
| ST26 / F24 Zhu et al., 2026 Reference 73 DOI 10.1186/s12951-026-04044-7 | Smoking-related MCI human fecal microbiome/metabolite association; aged male C57BL/6J chronic cigarette-smoke exposure model; antibiotic/FMT microbiota-transfer model; oral and intranasal *A. muciniphila*-OMV interventions; HMC3 microglia/SH-SY5Y neuron co-culture, CSE injury, AhR-inhibition and microglial metabolic assays | Native *A. muciniphila* ATCC BAA-835 OMVs containing ILA; purified ILA comparator; *A. muciniphila*-conditioned medium/fractionation and *E. coli* negative-control comparisons | Smoking-related MCI and smoke-exposed aged mice showed *A. muciniphila*/ILA depletion linked to cognitive impairment; *A. muciniphila*-OMVs contained ILA, reached brain tissue 24 h after intragastric gavage and localized to hippocampal IBA1+ microglia; oral or intranasal OMVs improved NOR/Y-maze cognitive readouts, restored microglial activation/synaptic-integrity markers and reduced neuroinflammatory cytokines; OMVs/ILA protected SH-SY5Y neurons from CSE-activated microglial toxicity, shifted microglial metabolism toward OXPHOS/ATP restoration, and AhR inhibition abolished these effects in vitro | Mostly aged male mice; FMT implicates smoke-altered microbiota but not selective *A. muciniphila* depletion; AhR causality shown in vitro but not with microglia-specific AhR knockout in vivo; OMV cargo causality focused on ILA but other cargo may contribute; no EV-depleted supernatant, heat-inactivated OMV, cargo-depletion/rescue, quantitative PK, immunogenicity or GLP repeat-dose toxicology |
| ST27 / F25 Hou et al., 2026 Reference 44 DOI 10.1007/s11427-025-3240-8 | Male C57BL/6J mice with ovalbumin-induced allergic asthma; bone-marrow-derived macrophage assays. | Native *A. muciniphila* OMVs and a noncovalent polymyxin-B-treated OMV construct; polymyxin B alone was tested. | Local OMV treatment reduced airway and type-2 inflammatory endpoints; the PMB-treated construct produced larger effects and lower LAL activity, with macrophage-state and AP-1/PI3K/AKT associations. | Randomization, blinding and sample-size justification were not reported. Omics and protein changes did not establish AP-1/PI3K/AKT necessity. DIR imaging tracked a lipophilic label rather than intact OMVs. |
| ST28 / F26 Pan et al., 2026 Reference 84 DOI 10.1002/adma.202515901 | Mucus and epithelial transport assays; CT26 cells; orthotopic CT26 colorectal tumours in male BALB/c mice. | Sucrose-gradient-purified *A. muciniphila* membrane vesicles electroporated with PD-L1 siRNA, with or without alginate-microgel encapsulation. | The construct improved siRNA stability, mucosal/tumour accumulation and PD-L1 knockdown and reduced tumour growth relative to free siRNA and liposomal delivery. | Efficacy and biodistribution groups were small (often n=3), blinding and sample-size justification were not reported, and a scrambled-siRNA control was not identified. Fluorescent siRNA recovery establishes cargo-label fate, not intact-carrier exposure. |
| ST29 / F27 Guan et al., 2026 Reference 67 DOI 10.1038/s41413-026-00541-5 | MR analysis plus human IVDD cohorts; microbiota-depleted, tail needle-puncture, natural-aging and bipedal-standing mouse IVDD models; human NP cells under TNF-alpha; microdissected NP/AF transcriptomics | Native *Akkermansia muciniphila* EVs from *Akk* BNCC341917; EV-enriched B2UKX5 effector protein; recombinant B2UKX5; *E. coli*/*E. coli*-EV comparators | Higher intestinal *Akk* abundance was inversely associated with IVDD risk/severity; *Akk* protection was abolished by GW4869-mediated EV-secretion inhibition; *Akk*-EVs localized to discs and recapitulated protection across injury, aging and mechanical-overload models; proteomics nominated B2UKX5, and recombinant B2UKX5 reproduced key anti-degenerative effects with NP/AF transcriptomic regulation | Clinical/MR data support association rather than therapeutic efficacy; GW4869 is pharmacologic and not EV-cargo-specific; B2UKX5 sufficiency shown but B2UKX5-depleted EV or cargo-loss/rescue experiments were not evident; exact ultracentrifugation g/time deferred to cited protocol/supplement; no GLP toxicology or PK package |
| ST30 / F16 Zhang et al., 2026 Reference 50 DOI 10.3389/fmicb.2026.1821362 | AOM/DSS colitis-associated CRC model combined with 14-day CUMS chronic-stress protocol in male C57BL/6J mice; HCT-116 and HT-29 CRC cell assays using ISO as an adrenergic stress mimic | Native *A. muciniphila* ATCC BAA-835 outer membrane vesicles (OMVs); *E. coli* OMV comparator; recombinant mouse Fetuin-A rescue | Chronic stress/ISO promoted CRC proliferation, migration, invasion and tumor burden while reducing cellular OMV uptake; *A. muciniphila* OMVs antagonized stress-driven CRC progression in cells and mice, downregulated Fetuin-A, and exogenous Fetuin-A partially reversed the OMV-associated benefit | Preclinical stress-CRC model; Fetuin-A rescue supports pathway involvement but does not identify the upstream OMV cargo; in vivo biodistribution, PK, systemic cytokines and formal repeat-dose toxicology were not evident; some culture centrifugation text contains redundant cell-removal steps requiring author/PDF check |
| ST31 / F28 Zhang et al., 2026 Reference 46 DOI 10.1097/fbp.0000000000000864 | Fourteen-month-old male C57BL/6J mice exposed to sevoflurane; cognitive, hippocampal inflammatory and colonic barrier readouts. | Native *AKK*-EV preparation. | Oral treatment improved cognitive and inflammatory endpoints and coincided with lower NLRP3, ASC and caspase-1 expression. | NLRP3 signalling was measured but not perturbed, so pathway necessity was not established. No exposure, cargo or active-comparator evidence was provided. |
| ST32 / F29 Ghaderi et al., 2025 Reference 37 DOI 10.18502/ijm.v17i6.20355 | In vitro human intestinal epithelial-barrier model using Caco-2 cells; comparison of active *A. muciniphila*, heat-inactivated *A. muciniphila*, cell-free supernatant and *A. muciniphila* OMVs; qRT-PCR readout of ZO-1 and occludin mRNA after 24 h exposure | Native *A. muciniphila* ATCC BAA-835 OMVs stored in 3% sucrose; active and heat-inactivated bacteria and cell-free supernatant used as comparator derivatives | *A. muciniphila* identity was confirmed by 16S rRNA PCR; OMVs showed TEM morphology with reported 30-300 nm diameter range and SDS-PAGE protein bands; active bacteria at MOI 10 and 50 increased ZO-1 mRNA, whereas heat-inactivated bacteria at MOI 50 decreased ZO-1; cell-free supernatant 7% increased both ZO-1 and occludin; OMVs did not significantly change ZO-1 but 50 ug/mL OMVs significantly increased occludin mRNA compared with sucrose control, while 100 ug/mL OMVs had no occludin effect | In vitro Caco-2 mRNA-only study; no TEER/permeability assay, immunoblotting, immunofluorescence localization, protein-level tight-junction validation, animal model, disease model or clinical samples; OMV preparation involved EDTA/deoxycholate extraction and lacks NTA, endotoxin/sterility testing, EV-depleted supernatant, heat-inactivated OMV, detergent-buffer control, cargo/receptor loss-of-function and formal safety assessment |
| ST33 / F32 Hamza et al., 2025 Reference 85 DOI 10.1016/j.apsb.2025.03.014 | In vitro 4T1 murine mammary carcinoma, MDA-MB-231 human breast cancer, and CT26-Luc cells; in vivo female BALB/c mice with subcutaneous 4T1 breast tumours and an orthotopic CT26-Luc intestinal/rectal tumour model. | siAtox1/ES@OMV, an engineered *Akkermansia muciniphila*-derived OMV construct co-loaded with Atox1 siRNA and elesclomol; single-cargo controls included siAtox1@OMV and ES@OMV. | The construct increased tumour delivery, Atox1 knockdown/copper retention and cuproptosis-associated readouts, promoted antitumour immune infiltration, and suppressed tumour growth in both subcutaneous 4T1 and orthotopic CT26-Luc mouse models. | The active intervention is a multi-component engineered OMV-siRNA-drug system; the independent contribution of native *AKK*-OMV biology, Atox1 knockdown, elesclomol loading and OMV immune stimulation remains difficult to separate, and long-term repeat-dose toxicology is not established. |
| ST34 / F33 Zhang et al., 2024 Reference 38 DOI 10.3389/fmolb.2024.1418876 | LS174T human goblet-like intestinal epithelial cells exposed to indomethacin. | *Akkermansia muciniphila*-derived outer-membrane vesicles (*AKK*-OMVs). | *AKK*-OMVs were reported to alleviate indomethacin-associated loss of mucin secretion and endoplasmic-reticulum stress. | A cell-line study does not establish functional barrier recovery, in vivo efficacy, product comparability, or safety. |
| ST35 / F31 Yaghoubfar et al., 2021 Reference 75 DOI 10.1007/s12602-021-09786-4 | Differentiated Caco-2 human colorectal adenocarcinoma epithelial monolayers. | *A. muciniphila* and *F. prausnitzii* EV preparations, compared with corresponding bacteria and PBS. | EV preparations increased extracellular serotonin and altered serotonin-system transcripts after 24 h. | Tumour-derived epithelial cell model; TEM-only EV characterization; no vesicle-dependency, cargo assignment, functional motility/barrier endpoint or organism-level validation. |
| ST37 / F12 Olarinoye et al., 2024 Reference 76 DOI 10.1038/s41598-024-69757-x | Spontaneously hypertensive (SHR) and Wistar-Kyoto (WKY) rats after intravenous vesicle administration. | *A. muciniphila*-derived extracellular vesicles (AmEVs). | Renal differential gene-expression responses were reported after intravenous AmEV administration. | Transcriptomic response does not by itself prove intact-vesicle renal delivery, organ-specific action, or therapeutic efficacy. |
| ST38 / F34 Zhu et al., 2025 Reference 51 DOI 10.1096/fj.202403295rr | SW480 and HT-29 colorectal-cancer cells with CD8 T-cell co-culture; reported SW480 xenografts in male BALB/c mice. | *A. muciniphila* OMVs and recombinant Amuc_1434 as separate interventions. | OMVs and recombinant Amuc_1434 reduced PD-L1-related and tumour-growth readouts and increased CD8-associated responses; co-immunoprecipitation supported interaction of recombinant Amuc_1434 with PD-L1. | No Amuc_1434-depleted OMV or cargo rescue was tested. The uptake assay used HT22 neuronal cells rather than the stated colorectal lines. The reported human SW480 xenograft in ordinary BALB/c mice and CD8 interpretation require clarification because immune status was not specified. |
| ST39 / F35 Tong et al., 2023 Reference 86 DOI 10.1016/j.mtbio.2023.100845 | Preclinical tumor-immunotherapy model using a multicomponent nanovaccine. | Hybrid construct combining tumor-cell exosomes, *Akk*-OMVs, liposomes, and a PD-L1 plasmid payload. | The engineered hybrid was reported to enhance local cancer immunotherapy, with *Akk*-OMVs serving as a natural-adjuvant component. | Efficacy cannot be attributed to native *Akk*-OMVs alone because the construct contains multiple vesicular and nucleic-acid components. |
| ST40 / F36 Kwak et al., 2026 Reference 58 DOI 10.1016/j.phrs.2026.108184 | C57BL/6 mice on a high-fat, methionine-low MASH diet; hepatic, metabolic and gut-microbiome endpoints; in vitro lipid-accumulation model. | PEG-precipitated *A. muciniphila* EV-enriched preparation compared with live bacteria. | The EV-enriched preparation reduced selected hepatic lipid and transcriptional endpoints. The authors explicitly acknowledged co-enrichment of non-vesicular material and absence of in vivo tracking. | PEG precipitation can co-isolate soluble proteins and macromolecules; marker and orthogonal-purity validation were absent. Live bacteria and particles were dosed in non-equivalent units, administration frequency and sex were not clearly reported, and no hepatic exposure was measured. |
| ST41 / F37 Zeng et al., 2026 Reference 68 DOI 10.1186/s12951-025-03948-0 | Vascular-calcification models with intermittent-fasting and mechanistic studies | *A. muciniphila*-derived EV preparation; B2URF3 studied as an enriched mediator | *AKK*-EVs were taken up by vascular smooth-muscle cells; B2URF3 was linked to ALDH1B1 and calcification readouts | The wider fasting-microbiome causal chain and contribution of other organisms are not fully excluded |
| ST42 / F38 Xu et al., 2026 Reference 71 DOI 10.1016/j.fbio.2026.108904 | Male C57BL/6J mice with MK-801-associated depressive-like and cognitive phenotypes; hippocampal, barrier, microbiota and short-chain-fatty-acid readouts. | Ultracentrifugation-enriched *A. muciniphila* EV preparation; DiR-labelled preparation used for distribution imaging. | Daily oral treatment improved behavioural and hippocampal inflammatory/synaptic readouts and altered gut microbial and metabolite measures. DiR signal was detected in cranial and excised brain regions at 24 h. | Outcome-specific n=3-8; randomization, blinding and sample-size justification were not reported. Lipophilic-dye fluorescence does not establish intact-vesicle passage across the blood-brain barrier, and no quantitative PK or cargo-dependency test was performed. |
| ST43 Reference 27 DOI 10.1002/advs.202523067 Source: Full text | BMSCs/RAW264.7; ovariectomized mice; human plasma EVs and bone measures. | EV cargo discovery; recombinant-protein intervention; human association | Amuc_1473 supported bone formation and limited osteoclastogenesis. Plasma EV-associated Amuc_1473 correlated with bone measures. | Supports cargo selection and biomarker research, not clinical efficacy of administered *AKK*-EVs. Do not transfer recombinant-protein efficacy/safety to intact EVs. Human evidence is observational. |
| ST44 Reference 70 DOI 10.4014/jmb.2507.07001 Source: Full text | C2C12 differentiation and dexamethasone atrophy. Separate SAMP8 animal arm used live bacteria. | Isolated *AKK*-EVs in vitro; live bacteria in vivo | EVs increased myogenic differentiation and protein synthesis and reduced dexamethasone-associated myotube atrophy. | A cellular rationale for muscle-directed development; animal sarcopenia benefit belongs to live bacteria. EV pharmacokinetics and animal efficacy not tested; supplementary experiment described in main text, not independently extracted. |
| ST46 Reference 87 DOI 10.1002/advs.202412434 Source: Full text | 4T1 breast tumors in female BALB/c mice; EMT-6 combination studies; four human tumor donors for metal mapping. | Engineered hybrid OMV drug delivery | Copper depletion inhibited tumor growth and altered immune/metabolic readouts; combination with anti-PD-1 improved response. | Evidence for an engineered delivery product, not unmodified *AKK*-EV monotherapy or liver-disease efficacy. Separate hybrid, chelator and targeting contributions. Human tissue mapping is not a treatment trial. |
| ST48 Reference 39 DOI 10.1007/s10123-023-00398-2 Source: Abstract only | Caco-2 exposed to toxigenic C. difficile RT001 supernatant. | Isolated *AKK*-EVs in vitro | EV-treated cells retained >90% viability versus untreated controls; bacterial preparations modulated toxin-associated cytokine/barrier-gene responses. | Supports epithelial compatibility and mechanistic testing, not prevention/treatment of clinical C. difficile infection. Full text needed for EV-specific effect sizes, timing, replicates and separation of results from other preparations. Online 2023; issue 2024. |
| ST50 Reference 35 DOI 10.1126/sciadv.ade5079 Source: Full text | Female BALB/c DSS colitis/gut disorder; CT26 tumors; epithelial and immune cells. | Native *AKK* OMVs; mucosal and tumor-combination experiments | Improved colitis readouts, mucus/tight-junction measures and IgA responses; enhanced anti-PD-1-associated tumor control. | Direct oral mucosal evidence; tumor benefit is combination evidence. DSS induction differs by experiment (7 versus 10 days). Particle tracking is not intact-particle pharmacokinetics. Outlier removal uses Dixon criterion. |
| ST51 Reference 59 DOI 10.1038/s41598-022-13840-8 Source: Full text | Caco-2 and HepG2 cells. | Chemically extracted membrane-vesicle preparation in vitro | ECS-related and PPAR transcripts changed in a treatment-, concentration- and cell-dependent manner. | Mechanistic metabolic-gene evidence, not demonstrated liver-disease benefit. Preparation differs from spontaneously released culture-supernatant EVs. No functional metabolic endpoint; unit/typographical inconsistencies in source retained as uncertainties. |
| ST53 Reference 69 DOI 10.1002/advs.202004831 Source: Full text | Ovariectomized C57BL/6 female mice; BMSCs/RAW264.7. | Native *AKK* EVs; distinct from mixed-microbiota EVs | *AKK* EVs improved bone mass/strength and formation–resorption readouts in OVX mice. | Supports oral extraintestinal biological activity; neither fracture prevention nor human efficacy established. Mixed-microbiota and pure-*AKK* interventions must remain separate. Fluorescent tissue signals alone do not quantify intact vesicle exposure. |
| ST54 Reference 74 DOI 10.1038/s41598-020-79171-8 Source: Full text | Male C57BL/6J mice, 8 weeks old; Caco-2 monolayers. | Native *AKK* EVs in healthy mice/cells | Colonic/hippocampal serotonin and pathway transcripts changed; cell serotonin did not significantly increase despite Tph1 induction. | Gut–brain signaling hypothesis from non-diseased animals; not evidence of treating depression. BBB crossing and survival through the gut were not directly demonstrated. Source filtration unit appears inconsistent. |
| ST55 Reference 55 DOI 10.1080/19490976.2017.1405209 Source: Full text | Aged, HFD-fed male C57BL/6N mice. | Native *AKK*-EV intervention distinct from metformin/FMT | Weight gain 0.5±1.5 versus 3.0±1.2 g; cholesterol lower. Serum glucose unchanged; fat-pad difference not significant (P=0.057). | Small metabolic pilot with null glycemic result; not an established antidiabetic EV treatment. Unresolved duration and HFD-timing discrepancy between methods and Fig. 5; limited precision from small groups. |

# Supplementary Table S3 Strains, vesicle preparation and dosing

Reported dose units and route-specific schedules are retained. Inconsistent or unavailable details remain explicit; particle, protein and cargo doses are not interchangeable. This table supports comparison of the preparations used in Table S2.

| **Study** | **Strain/clade reported** | **EV isolation/engineering method** | **Characterization methods** | **Dose metric** | **Route/frequency** |
| --- | --- | --- | --- | --- | --- |
| ST01 | *A. muciniphila* ATCC BAA-835 reported; clade not specified | Anaerobic culture at 37 C under 99% N2 to OD600 1.5; 10,000 g pelleting, 0.45/0.22 um filtration, QuixStand enrichment, 150,000 g ultracentrifugation for 2 h at 4 C | TEM, dynamic light scattering, SDS-PAGE/Coomassie, fecal EV 16S rDNA metagenomics, FITC-dextran permeability, TEER, western blotting, H&E | In vivo AmEV 10 ug/mouse orally every other day for 2 weeks; Cy7-AmEV 50 ug total protein for distribution; Caco-2 AmEV 0.1, 1, 10 ug/mL; LPS 5 ug/mL | Oral administration/gavage after 10-12 weeks HFD; every other day for 2 weeks; cell assays usually 4 h exposure |
| ST02 | *A. muciniphila* MucT/ATCC BAA-835 purchased from DSMZ; clade not specified | *A. muciniphila* cultured anaerobically in basal mucin-based medium then BHI plus 0.5% mucin; bacterial cultures pelleted at 10,000 g for 20 min; EVs isolated by ultracentrifugation at 150,000 g for 2 h at 4 C; pellets resuspended in 3% sucrose and stored at -80 C | SEM morphology/size range, Bradford protein assay, trypan-blue viability assay, qRT-PCR for TLR2, TLR4, OCLN, ZO1, ZO2, ZO3 and CLDN4, GAPDH normalization | Live *A. muciniphila* MOI 10; EV treatments reported as 0.1, 0.5 and 5 ug in methods/figures, with results text also describing ug/mL concentrations; 24 h exposure | In vitro treatment for 24 h; no in vivo route/frequency |
| ST03 | *A. muciniphila* MucT/ATCC BAA-835 from DSMZ; clade not specified | *A. muciniphila* cultured anaerobically in basal mucin-based medium then BHI plus 0.5% mucin; supernatant filtered; EVs isolated by ultracentrifugation at 200,000 g for 2 h at 4 C; pellets resuspended in PBS and stored at -80 C | SEM morphology, SDS-PAGE protein pattern, LAL chromogenic endotoxin/LPS assay, plasma glucose/TC/TG kits, H&E histology, qRT-PCR for adipose and colon genes, Caco-2 viability and inflammatory cytokine multi-analyte ELISA | In vivo live *A. muciniphila* 10^9 CFU/200 uL; EVs 10 ug protein/200 uL; Caco-2 live *A. muciniphila* MOI 100 and EVs 10 ug for 24 h | Oral gavage for 5 weeks after 3 months HFD induction; exact gavage frequency not explicit in extracted XML; Caco-2 treatment 24 h |
| ST04 | *A. muciniphila* MucT/ATCC BAA-835; clade not specified | *A. muciniphila* cultured in basal mucin-based medium; after OD600 reached 1, pellets were removed and supernatant filtered; EVs isolated by ultracentrifugation at 200,000 g for 2 h at 4 C; pellets resuspended in PBS and stored at -80 C; pasteurization performed at 70 C for 30 min | SEM morphology/size range 40-150 nm, SDS-PAGE protein profile, plasma glucose/TC/LDL/HDL/TG/ALT/AST, plasma and eWAT TNF-alpha/IL-6/IL-10 ELISA, H&E histology of liver/colon/eWAT, qRT-PCR for liver/colon/adipose genes and stool microbiota qPCR | Live *A. muciniphila* 10^9 CFU/200 uL; pasteurized *A. muciniphila* 10^9 CFU/200 uL; EVs 10 ug protein/200 uL; HFD 60% energy from butter for 5 weeks | Treatment given for 5 weeks with HFD; exact route and dosing frequency are not explicit in extracted XML; body weight measured weekly and food monitored daily |
| ST05 | *A. muciniphila* MucT/ATCC BAA-835 from DSMZ; clade not specified | *A. muciniphila* cultured anaerobically in basal mucin-based medium then BHI plus 0.5% mucin; supernatant filtered through 0.22 um membrane; EVs isolated by ultracentrifugation at 200,000 g for 2 h at 4 C; pellets resuspended in sterile PBS and stored at -80 C; pasteurized bacteria prepared at 70 C for 30 min | SEM morphology/size 40-150 nm, SDS-PAGE protein bands 11-245 kDa, LX-2 qRT-PCR, cell viability assay, serum ALT/AST/glucose/lipids and TNF-alpha/IL-6/IL-10 ELISA, H&E and Masson's trichrome, hepatic alpha-SMA western blot, tissue qRT-PCR, fecal targeted microbiota qPCR | In vivo live/pasteurized *A. muciniphila* 10^9 CFU/200 uL; EVs 50 ug protein/200 uL; HFD plus 10% CCl4 2 mL/kg intraperitoneally twice weekly; LX-2 live/pasteurized bacteria MOI 1/10/100 and EVs 1/10/50 ug/mL after LPS 0.01 ug/mL activation | Daily oral gavage for 4 weeks; CCl4 intraperitoneal injection twice weekly for 4 weeks; LX-2 activation 6 h followed by 24 h treatment |
| ST06 | *A. muciniphila* MucT/ATCC BAA-835 from DSMZ; clade not specified | *A. muciniphila* cultured in BHI broth plus 0.5% mucin; bacterial suspension centrifuged at 11,000 g for 20 min; pasteurized bacteria heated at 70 C for 15 min; EVs isolated from culture supernatant by ultracentrifugation at 200,000 g for 2 h at 4 C | LX-2 qRT-PCR for TLR5/TLR9 and PPAR-alpha/gamma, H&E histopathology and blinded histometric scoring, colon OCLN/CLDN1/CLDN2 qRT-PCR, liver/adipose cytokine ELISA, tissue qRT-PCR for inflammatory and anti-inflammatory genes | In vivo live/pasteurized *A. muciniphila* 10^9 CFU/200 uL; EVs 50 ug protein/200 uL; HFD plus 10% CCl4 2 mL/kg intraperitoneally twice weekly; LX-2 live/pasteurized bacteria MOI 1/10/100 and EVs 1/10/50 ug/mL after LPS 0.01 ug/mL activation | Daily oral gavage for 4 weeks; CCl4 intraperitoneal injection twice weekly for 4 weeks; LX-2 activation 6 h followed by 24 h treatment |
| ST07 | *A. muciniphila* ATCC BAA-835 reported; clade not specified | *Akk* cultured anaerobically at 37 C in BHI with 0.5% porcine mucin and 0.05% L-cysteine; culture medium centrifuged twice at 6,000 g for 30 min at 4 C, 0.45 um filtered, concentrated with 100 kDa Amicon Ultra-15, separated by OptiPrep density-gradient ultracentrifugation at 100,000 g for 18 h at 4 C, resuspended in PBS, 0.22 um filtered and stored at -80 C | TEM, DLS particle sizing, total protein quantification, tumor-volume/weight and Ki67 staining, flow cytometry for CD8/GZMB/IFN-gamma and macrophage markers, qRT-PCR, CD86 immunofluorescence, CCK-8, colony formation, scratch wound healing, H&E organ histology | In vivo *Akk*-EVs 40 ug per mouse; in vitro macrophage treatment 10 ug/mL for 24 h; CD8/macrophage and normal-cell assays used study-specific exposure ranges | Tail-vein injection every other day starting 5 days after RM-1 inoculation; treatment/observation lasted 13 days before sacrifice; RM-1 cells were implanted subcutaneously in the right axilla |
| ST08 | *A. muciniphila* ATCC BAA-835; clade not specified | *Akk* cultured in modified basal mucin-based medium at 37 C under 80% N2/10% H2/10% CO2 for 3-5 days to OD600=1; culture broth sequentially centrifuged at 3000 g and 10,000 g for 10 min each; supernatant filtered through 0.22 um sterile filters; EVs pelleted by twice ultracentrifugation at 135,000 g, 4 C for 70 min in SW32 Ti rotor; final pellets resuspended in PBS, quantified by BCA and stored at -80 C | TEM, NTA, SDS-PAGE/Coomassie, western blot for OmpA/LPS, PKH26 biodistribution imaging, CellMask trophoblast uptake/confocal imaging, SBP and urine-protein assays, H&E/PAS, CK7/alpha-SMA IHC, foetal/placental weights, placental RNA-seq, RT-qPCR, western blot for EGFR/p-PI3K/PI3K/p-AKT/AKT, HTR-8/SVneo proliferation and wound-healing assays, 16S/RT-qPCR microbiota readouts and natural fertility assay | Live Am 200 uL of 1 x 10^8 CFU/mL; AmEVs 20 ug per mouse or 20 ug/200 uL for biodistribution; L-NAME 125 mg/kg/day by subcutaneous injection; HTR-8/SVneo AmEV pretreatment 1, 10, 20 or 50 ug/mL for 24 h; labelled AmEV uptake incubation 4 h | Oral Am or AmEVs every other day for 2 weeks before mating and continued through E17.5; L-NAME from E10.5 for 8 days; SBP measured baseline/E0.5/E4.5/E8.5/E12.5/E16.5; urine at premating and E17.5; biodistribution imaged 5 h after gavage; HTR model used 8 h hypoxia plus 16 h reoxygenation |
| ST09 | *A. muciniphila* ATCC BAA-835 provided by China Agricultural University; clade not specified | *Akk* cultured anaerobically in BHI plus 0.05% L-cysteine-HCl; bacterial liquid centrifuged 10,000 g for 20 min at 4 C; supernatant filtered through 0.22 um PES filter, concentrated by Vivaflow 50 crossflow cassette, ultracentrifuged at 200,000 g for 3 h at 4 C, washed by 200,000 g for 1.5 h, resuspended in PBS and stored at -80 C | TEM, NTA, DiI-labeled EV uptake/confocal imaging, RAW264.7 NO/cytokine assays, DSS DAI/body weight/colon length, H&E injury scoring, AB-PAS mucus staining, IHC for MUC2/ZO-1, ELISA for IgA/sIgA/MPO/TNF-alpha/IL-1beta/IL-6/DAO/D-LA, qRT-PCR and 16S rRNA V3-V4 sequencing | In vivo *Akk* 10^9 CFU in 0.2 mL PBS; *Akk* EVs 10 ug protein in 0.2 mL PBS; DSS 3.5% in drinking water days 22-28; RAW264.7 DiI-EVs 100 ug/mL for uptake; LPS 100 ng/mL with *Akk* EVs 10, 50 or 100 ug/mL or *Akk* 10^7 CFU | Oral gavage once daily from days 8-28; DSS induction days 22-28; RAW264.7 LPS stimulation followed by 24 h EV treatment; sacrifice day 29 |
| ST10 | *A. muciniphila* MucT (ATCC BAA-835) from DSMZ; clade not reported. | Mucin-based then BHI plus 0.5% mucin culture; 6,000 x g; 0.22 micrometre filtration twice; 130,000 x g for 2 h; PBS wash and resuspension. | TEM, NanoDrop protein estimate and SDS-PAGE protein profile. | OMVs 50 micrograms/mL; live bacteria MOI 50 and 100; LPS 200 ng/mL; dexamethasone 1 micromolar. | Overnight in vitro exposure on day 5 of dendritic-cell differentiation. |
| ST11 | *A. muciniphila* ATCC BAA-835/DSM 22959/KCTC 15667; clade not reported. | 6,000 x g for 20 min; 0.22 micrometre filtration; 100,000 x g for 2 h; PBS resuspension; storage at -80 C. | TEM, NTA and SDS-PAGE/silver-stain protein profiling. | 1 x 10^8 or 1 x 10^9 particles/kg. | Tail-vein injection once weekly for 4 weeks; direct ex vivo aortic-ring exposure in a separate assay. |
| ST12 | *A. muciniphila* ATCC BAA-835; clade not reported. | Harvested bacterial cells were sonicated, centrifuged at 11,000 x g, filtered at 0.22 micrometres and ultracentrifuged at 120,000 x g for 2 h, then PBS-washed. | TEM, NTA and BCA protein assay. | Group description states 100 micrograms protein/200 microlitres; later methods state 2 x 10^8 particles/0.1 mL. | Daily oral administration for 4 weeks after surgery; PLX5622 chow for microglial ablation. |
| ST13 | *A. muciniphila* MucT (ATCC BAA-835) from DSMZ; clade not reported. | Culture-supernatant ultracentrifugation at 200,000 x g for 2 h; PBS resuspension; upstream clarification/filtration details were not reported. | No TEM, NTA/DLS, marker, purity or batch data reported for the administered preparation. | EVs 10 micrograms protein/200 microlitres; live or pasteurized bacteria 10^9 CFU/200 microlitres. | Four-week intervention; route and frequency were not clearly reported in the methods text. |
| ST14 | *A. muciniphila* MucT (ATCC BAA-835) purchased from Shanghai Biological Specimen Conservation Centre; clade not specified | *Akk* cultured anaerobically at 37 C in BHI broth supplemented with 0.5% mucin; bacterial suspension centrifuged at 4410 g for 10 min at 25 C; supernatant filtered through 0.45 um filter; ultracentrifuged at 120,000 g for 70 min at 4 C; pellet resuspended in sterile PBS and washed by repeating centrifugation twice; final pellet weighed, resuspended in 1 mL PBS and stored at -80 C | Cryo-TEM/JEM-1200EX, Malvern ZS90 particle-size analysis, BCA protein assay, SDS-PAGE, HPLC-MS proteomics, Cello2GO subcellular localization, GO/KEGG enrichment, Caco-2 MTT viability, NO/ROS/MDA/CAT assays, qRT-PCR for TNF-alpha/IL-1beta/IL-6, RNA-seq/edgeR/STRING/Cytoscape and western blot for TRIF/MyD88/p38 MAPK/FOS/TGFBR2 | Caco-2 LPS screening 0.1, 1, 10 and 100 ug/mL; inflammatory model used LPS 10 ug/mL for 18 h; Am-EV testing 1, 25 and 50 ug/mL for 12 h after LPS, with broader viability screening up to 100 ug/mL; Am-EV concentration reported as 14.50 +/- 2.23 mg/mL | Caco-2 cells treated with LPS for 18 h followed by Am-EVs for 12 h; transcriptome group used 10 ug/mL LPS for 18 h then 50 ug/mL Am-EVs for 12 h; EV storage at -80 C after isolation |
| ST15 | *A. muciniphila* ATCC BAA-835 from DSMZ; clade not reported. | 11,000 rpm for 20 min; 0.45 micrometre filtration; 200,000 x g for 2 h; PBS resuspension; 0.22 micrometre final filtration; storage at -80 C. | TEM, SEM, SDS-PAGE, DLS and NanoDrop absorbance; NanoDrop is not a purity-specific vesicle assay. | EVs 50 and 100 micrograms/mL; bacterial exposure is inconsistently reported across methods and results. | In vitro exposure for 24 h. |
| ST16 | *A. muciniphila* ATCC BAA835 from China BeNa Cultural Collection; clade not specified | *Akk* cultured anaerobically at 37 C in BHI with 0.05% mucin under 10% H2/10% CO2/80% N2 for 4 days; AOMVs obtained by differential centrifugation: 10,000 g for 60 min at 4 C, 0.22 um filtration, ultracentrifugation at 200,000 g for 3 h, pellet resuspended in 200 uL PBS and protein quantified by BCA; DiD-labelled vesicles ultracentrifuged at 200,000 g for 3 h to remove unbound dye | Milk SCC/CMT and 16S rDNA sequencing, bovine/mouse cytokine ELISAs, MAC-T CCK-8 viability, qRT-PCR/inflammatory cytokine assays, TEM, NanoSight/Flow Nano Analyzer particle sizing, BCA protein/particle counting for GW4869 secretion inhibition, DiD confocal uptake and in vivo fluorescence imaging, H&E histopathology/scoring, western blot for TLR4/p-I-kappaB/I-kappaB/p-p65/p65 | MAC-T screening: LPS 1-200 ug/mL, LA/PA 1 x 10^6 to 1 x 10^9 CFU/mL, AOMVs 10-100 ug/mL; main MAC-T conditions used LA/PA 1 x 10^7 CFU/mL, AOMVs 50 ug/mL and LPS 50 ug/mL; mouse AOMV treatment 50 ug/kg by tail vein; mouse mastitis induced with 50 uL LPS 0.2 mg/mL trans-nipple; cow live-*Akk* supplementation 1 x 10^11 CFU in 40 mL daily | MAC-T cells pretreated with *A. muciniphila*/AOMVs for 4 h then LPS for 24 h; DiD-AOMV distribution measured 12 h after tail-vein injection; mouse AOMV given 12 h before LPS mastitis induction and mammary glands collected 24 h after LPS; cow live-*Akk* supplementation daily at 06:30 for 3 consecutive weeks |
| ST17 | *A. muciniphila* ATCC BAA-835 purchased from Mingzhou Bio Co., Ltd.; clade not specified | *Akk* cultured anaerobically at 37 C for 72 h; supernatant centrifuged 5000 g for 10 min at 4 C, then 15,000 g for 20 min at 4 C and filtered through 0.45 um; OMVs pelleted at 170,000 g for 80 min at 4 C, washed by 170,000 g for 60 min, resuspended in sterile PBS, filtered through 0.45 um under aseptic conditions and stored at -80 C | NTA, TEM, simulated gastric/intestinal-fluid stability testing, SDS-PAGE/Coomassie, label-free LC-MS/MS proteomics, 16S rRNA sequencing, qPCR for *A. muciniphila* abundance, AOM/DSS/CUMS CAC phenotyping, tumor burden/macroscopy, Ki67 IHC, histopathological scoring, Dil-OMV confocal uptake, CCK-8 viability, colony formation and GMrepo/human tumor-tissue correlation analyses | Live *Akk* 1 x 10^9 CFU in 200 uL by oral gavage; OMVs 20 ug protein in 100 uL by oral gavage; AOM 10 mg/kg intraperitoneal day 0; DSS 2% for three 7-day cycles separated by 14-day water periods; CRC cell colony assays used 20 ug OMVs; Dil-labelled OMV uptake coculture 10 h | Live *Akk* or OMV gavage daily for 14 consecutive days; CUMS applied for 14 days; SGF/SIF stability assessed for 2 h and 4 h at 37 C; CRC cell proliferation measured after 2 days of treatment and colony formation after 15 days |
| ST18 | Multiple clades/strains reported: AmI EB-AMDK19 plus BAA-835/EB-AMDK7/EB-AMDK23; AmII EB-AMDK39/EB-AMDK43; AmIV included in population-level clade analysis | Culture supernatants filtered 0.22 um and concentrated with 500 kDa hollow-fiber QuixStand; enriched EVs prepared by 80,000 g for 1 h then 150,000 g for 3 h at 4 C and resuspended in 20 mM Tris-HCl pH 8.0; SEC on Sepharose CL-6B used for culture supernatant and fecal soluble fractions; fecal EVs extracted from 0.5 g feces via PBS extraction, 0.45/0.2 um filtration, 30 kDa concentration and SEC | NTA, TEM, SEC fraction profiling, protein quantification, disk diffusion, qPCR with clade/strain-specific primers, bacterial flow cytometry for IgA binding, heat/proteinase K/molecular-weight cutoff tests, and LC-MS/MS proteomics for EV-enriched proteins | In vitro AmII EV-to-AmI cell ratios 0.1-1000 against 1 x 10^6 CFU/culture with complete inhibition at >=100-fold; supernatant/fraction assays used 10-20% v/v; oral enriched EB-AMDK39 EVs 50 ug/mouse on days 0 and 4; live-strain challenges used 1 x 10^8 CFU AmI with AmII at 1/5 or 1/25 of AmI in IgA experiments | In vitro growth assays generally assessed after 24-48 h; live *A. muciniphila* strains and enriched EVs were administered orally/gavage to GF or gnotobiotic mice; feces collected longitudinally including days 7 and 14 after EV treatment and after day-14 strain challenge |
| ST19 | *A. muciniphila* obtained from State Key Laboratory of Food Science and Resources, Nanchang University; strain/clade not specified in extracted XML | *A. muciniphila* cultured anaerobically at 37 C for 48 h in BHI; culture centrifuged 12,000 rpm for 20 min; supernatant sequentially filtered through 1.2, 0.45 and 0.22 um filters; ultracentrifuged at 150,000 g for 2 h at 4 C; pellet resuspended in sterile PBS, quantified by BCA and stored at -80 C | TEM, NanoSight/NTA, BCA protein assay, PKH26 biodistribution/cellular uptake, DIA proteomics, Y-maze/NOR behavior, H&E/IHC/IF, qPCR, ELISA/Luminex cytokines, 16S rRNA sequencing, GC-SCFA analysis, untargeted plasma metabolomics, HT-22 cell assays | In vivo 200 uL AmEV suspension containing 20 ug EV protein by gavage; in vitro HT-22 LPS 1 ug/mL plus AmEVs 10 ug/mL; recombinant Amuc 1100 0.5 ug/mL; inhibitors 25 uM; PKH26-labeled AmEV biodistribution assessed at 6 h | Oral gavage during 21-day protocol; DSS 2.9% in drinking water from day 15; behavioral testing days 20-21; sacrifice day 22; HT-22 treatments co-incubated for 24 h and uptake assessed after 6 h |
| ST20 | *A. muciniphila* ATCC BAA-835 reported; clade not specified | *A. muciniphila* cultured anaerobically in mucin-based medium; supernatants sequentially filtered through 0.45 and 0.22 um membranes; ultracentrifuged at 150,000 g for 2 h at 4 C; EVs washed and resuspended in sterile PBS | TEM, NTA, fecal qPCR, OGTT/ITT/PTT, FBG/FINS/HOMA-beta, pancreatic and hepatic H&E, insulin western blot, ALT/AST/TG kits, MDA/GSH-Px/SOD/CAT assays, ELISA cytokines, pancreatic lymph-node flow cytometry and pancreatic CD4/Foxp3 immunofluorescence | STZ 50 mg/kg/day intraperitoneally for 5 days; oral AmEVs 10, 20 or 40 ug in 100 uL saline daily for 8 weeks; heat-inactivated *A. muciniphila* 10^5 CFU/day; anti-CD25 clone PC61 for Treg depletion with maintenance injections every 3 weeks | Daily oral gavage after hyperglycemia confirmation; 8-week intervention; metabolic tests after fasting with oral glucose 2 g/kg, insulin 0.75 U/kg intraperitoneally and pyruvate 2 g/kg intraperitoneally |
| ST21 | *A. muciniphila* species reported; strain/clade not specified in the extracted XML key methods; not verified from the available article text. | *A. muciniphila* cultured anaerobically at 37 C for 48 h; culture supernatant centrifuged at 10,000 x g for 30 min, filtered through 0.45/0.22 um membranes, concentrated with 100 kDa Centricon Plus-70, ultracentrifuged at 150,000 x g for 2 h at 4 C, washed in sterile PBS, refiltered through 0.22 um and stored at -80 C. Engineered SMB made by sequential extrusion of BMVs and siRNA-MOF cores through 400 nm and 200 nm membranes, followed by centrifugation and saline washing. | TEM, NTA, DLS size/zeta and stability, elemental distribution, powder XRD, UV-Vis spectra, release/ultrasound-response assays, uptake assays, flow cytometry, RNA-seq, western blotting, immunofluorescence and immunohistochemistry. | In vitro SMB 500 ug/mL in ultrasound-release/cell-exposure assays; siRNA loading range 50-500 nM; in vivo nanoformulation dose not reported from the article text used for this audit. | Tail-vein administration every 4 days in orthotopic HCC studies, with ultrasound 24-48 h after each injection (1.0 MHz, 2.5 W/cm2, 50% duty cycle, 5 min); H22 tumors treated after 14 days and assessed at day 22. |
| ST22 | *A. muciniphila* sourced as ATCC 107961 in official methods; clade not reported. | *AKK* culture supernatant centrifuged at 10,000 xg for 30 min, filtered through 0.22 um, concentrated with a 100 kDa MWCO device, and ultracentrifuged at 1.5 x 10^5 xg for 3 h; BT-*AKK*-EVs were generated using DSPE-PEG2000-Mal-Cys-SDSSD conjugation/hydrophobic insertion. | TEM, NTA, zeta potential, miRNA sequencing, Cy5 biodistribution imaging, BMSC/BMM assays, ALP, ARS, TRAP/F-actin staining, qRT-PCR and western blotting for PI3K-AKT/osteogenic/osteoclast markers. | Biodistribution used Cy5-labeled *AKK*-EVs or BT-*AKK*-EVs via tail vein at 1 ug/uL with 200 uL per mouse; in vitro BMSCs received 30 ug EVs and BMM osteoclastogenesis assays received 15 ug EVs. | Tail-vein injection for biodistribution and in vivo evaluation; imaging reported at 4 h post-injection. In vitro ALP assessed day 7, ARS day 14 and osteoclastogenesis day 7. |
| ST23 | *A. muciniphila* ATCC BAA-835 reported; clade not specified | Anaerobic culture in 90% N2/10% CO2; EVs isolated from culture media/stool/small intestinal fluids by ultracentrifugation at 200,000 g for 2 h at 4 C | TEM, nanoparticle tracking analysis, western blot, SDS-PAGE/protein profiling, 16S rDNA metagenomics/PCA, IL-6 assays, H&E histology | In vivo AmEV dose reported as 100 ug/mouse in Fig. 6 legend, while the results text says 100 mg/mouse; not verified from the available article text; in vitro AmEV 10 ng/mL, 100 ng/mL, 1 ug/mL | DSS in drinking water for 5 days; AmEV or live bacteria administered concomitantly with DSS; exact route/frequency of AmEV administration requires supplement/PDF confirmation |
| ST24 | *A. muciniphila* ATCC BAA-835; clade not reported. | BHI plus cysteine culture; 4,000 x g, 10,000 x g, 0.22 micrometre filtration and two 150,000 x g ultracentrifugation steps. | TEM, NTA, DLS, zeta potential, OmpA, SDS-PAGE, total RNA and miRNA sequencing. | Oral 1 x 10^10 particles/100 microlitres daily; subcutaneous 2 x 10^9 particles/20 microlitres daily. | Oral or subcutaneous once daily for 10 days. |
| ST25 | *A. muciniphila* BAA-835 (ATCC); clade not reported. | BHI plus mucin/cysteine; 4,000 x g for 30 min; 0.22 micrometre filtration; 100,000 x g for 90 min; PBS resuspension; storage at -80 C. | Protein profile/silver staining and lipophilic-dye uptake/localization; particle size and orthogonal purity data were limited in the main methods. | Oral live bacteria 3 x 10^8 CFU, EVs 50 micrograms or P9 50 micrograms per mouse, three times weekly. | Oral treatment for 8 weeks; separate DiR/DiO-labelled EV tracing by tail-vein injection with harvest at 3 h. |
| ST26 | *A. muciniphila* ATCC BAA-835; clade not specified | *A. muciniphila* cultured anaerobically at 37 C in modified basal mucin-based medium for 3-5 days until OD600 = 1.0; culture broth sequentially centrifuged at 3000 g and 10,000 g for 10 min each; supernatant filtered through 0.22 um sterile filters; OMVs pelleted by two rounds of ultracentrifugation in an SW32 Ti rotor at 135,000 g for 70 min at 4 C, resuspended in PBS, quantified by BCA protein assay and stored at -80 C | TEM, NTA, BCA protein assay, LC-MS/ELISA for ILA, DiD-labelled OMV biodistribution, hippocampal confocal microscopy/IBA1 colocalization, NOR/Y-maze behavior, 16S rDNA sequencing, UHPLC-MS/MS metabolomics, MoCA, FMT, CSE assays, ELISA/WB/qPCR for cytokines, SH-SY5Y/HMC3 co-culture, Seahorse ECAR/OCR, ATP/LDH/apoptosis assays and AhR inhibition with CH223191 | OMV intervention 1 ug/mouse by oral gavage or intranasal instillation; purified ILA 10 mg/kg tail vein in methods and/or intervention comparator; HMC3 OMVs 10 ug/mL and ILA 100 uM with 5% CSE; CH223191 10 uM; smoke exposure 1 cigarette/day at 25 mL/min for 12 weeks; FMT 200 uL of 200 mg/mL fecal suspension | Smoke exposure for 12 weeks; OMV/ILA interventions every other day for six doses after smoke exposure in results; methods report oral gavage or intranasal instillation for OMVs; DiD-OMV brain imaging 24 h after intragastric gavage; FMT over 28 days after 7-day antibiotics with a protocol-frequency discrepancy to verify; HMC3 AhR inhibitor 1 h pretreatment then 24 h CSE/OMV/ILA |
| ST27 | *A. muciniphila* BAA-835 (ATCC); clade not reported. | 4,000 x g; 0.22 micrometre filtration; 10,000 x g; 100-kDa ultrafiltration; 100,000 x g for 2 h. PMB incubation followed by 100,000 x g for 2 h to remove unbound material. | TEM, NTA, LAL endotoxin assay and SDS-PAGE protein profile; native and PMB-treated particles had similar mean size. | 50 microlitres of 10 micrograms/mL OMVs or PMB-treated OMVs per intratracheal administration (0.5 micrograms total protein). | Intratracheal dosing every 2 days before the first OVA challenge, then weekly during sensitization; locally administered DIR-labelled OMVs for distribution imaging. |
| ST28 | *A. muciniphila* ATCC BAA-835; clade not reported. | 12,500 x g for 20 min; 0.22 micrometre filtration; 200,000 x g for 2 h; 8-60% sucrose-gradient ultracentrifugation for 18 h; additional 200,000 x g wash. siRNA loaded by electroporation, followed by 100,000 x g removal of free cargo. | TEM, size, zeta potential, protein content, siRNA loading/encapsulation, fluorescence colocalization and stability assays. | 2 micrograms siRNA per mouse for in vivo distribution, efficacy and safety experiments. | Oral gavage every 3 days for efficacy and 30-day safety; tumour and intestinal distribution measured after oral dosing. |
| ST29 | *Akkermansia muciniphila* BNCC341917; clade not specified | Anaerobic culture at 37 C in BHI broth plus 0.05% L-cysteine-HCl; *Akk*-EVs isolated from culture supernatant by ultracentrifugation following an established protocol; GW4869 cultures used to suppress EV secretion before bacterial gavage | TEM cup-shaped morphology, NTA size/yield and GW4869 suppression, DiR EV tracing/IVIS in L2-6 discs, label-free proteomics, competitive ELISA for *Akk*-EVs/B2UKX5, MRI/DR/micro-CT, H&E/SO-FG, IF markers, qRT-PCR, cytokines/serum biochemistry, NP/AF transcriptomics | *Akk*/*E. coli* 1.5 x 10^8 CFU per dose orally 3 times/week; oral *Akk*-EVs/*E. coli*-EVs 100 ug per dose 3 times/week or tail-vein EVs 30 ug once/week; B2UKX5 30 ug tail-vein once/week in puncture model and 50 ug orally twice/week for 6 months in aging model; bipedal model used *Akk* 3.0 x 10^8 CFU or *Akk*-EVs 100 ug orally twice/week for 12 weeks | Oral gavage or tail-vein injection depending on experiment; tail puncture interventions 8 or 12 weeks; natural-aging intervention from 15 to 21 months; bipedal standing 8 h/day, 7 days/week for 12 weeks with twice-weekly oral intervention |
| ST30 | *A. muciniphila* ATCC BAA-835; 16S rRNA sequence provided in Supplementary Data Sheet S1; clade not specified | *A. muciniphila* cultured in chopped meat carbohydrate broth for 72 h at 37 C anaerobically; cells/debris removed by centrifugation and 0.22 um filtration; OMVs pelleted at 170,000 x g for 80 min at 4 C, washed in PBS under same conditions, resuspended, filter-sterilized and stored at -80 C; sterility checked on anaerobic agar plates | TEM, NTA, NanoPhotometer protein concentration, SDS-PAGE/Coomassie, anti-LPS western blot, sterility culture, DiI OMV uptake/confocal imaging, CCK-8, colony formation, Transwell migration/invasion, proteomics/KEGG/GO, ELISA, western blot and IHC for Fetuin-A | In vivo *A. muciniphila* OMVs 20 ug total protein in 100 uL PBS by daily oral gavage for 14 days; recombinant mouse Fetuin-A 50 mg/kg tail-vein injection once on day 1; in vitro OMVs 20 ug/mL for functional assays and 100 ug/mL DiI-labeled OMVs for uptake assays; ISO 20 uM | Daily oral gavage during 14-day CUMS/intervention period; AOM 10 mg/kg intraperitoneal once followed by three 2% DSS cycles; Fetuin-A single tail-vein injection on day 1; in vitro ISO exposure 48 h for migration/invasion/Fetuin-A and 14 days for colony formation |
| ST31 | *A. muciniphila* CICC 24917; clade not reported. | 3,000 x g, 10,000 x g, 0.22 micrometre filtration and two 135,000 x g ultracentrifugation steps; PBS resuspension; storage at -80 C. | TEM and NTA. | 2 x 10^8 particles per mouse. | Oral administration for 2 weeks after sevoflurane exposure. |
| ST32 | *A. muciniphila* ATCC BAA-835 obtained from DSMZ; clade not specified | *Akk* cultured anaerobically in BHI agar/broth supplemented with 0.5% mucin type III, 5 ug/mL hemin, 1 ug/mL menadione and 0.05% L-cysteine under 80% N2/10% H2/10% CO2 at 37 C; after overnight growth to approximately OD600 1.5, bacteria were centrifuged at 6000 g at 4 C, pellets washed twice with PBS, suspended in 9% NaCl, centrifuged at 6000 g at 4 C for 1 h, suspended in EDTA and sodium deoxycholate buffers, centrifuged at 20,000 g at 4 C for 1 h, then ultracentrifuged twice at 125,000 g for 2 h; OMVs stored in 3% sucrose at -70 C | 16S rRNA PCR for bacterial identity, TEM at 100 kV after negative staining with 2% phosphotungstic acid, ImageJ particle-size assessment, SDS-PAGE/Coomassie protein-band confirmation, Caco-2 qRT-PCR for ZO-1 and occludin normalized to GAPDH, RNA integrity/purity checks and GraphPad statistical analysis | Caco-2 cells seeded at 5 x 10^5 cells/well in six-well plates; active bacteria MOI 10, 50 and 100; heat-inactivated bacteria MOI 10, 50 and 100 after 56 C for 20 min; OMVs 50 and 100 ug/mL; cell-free supernatant 7% v/v; PBS, 3% sucrose and BHI controls | Single 24 h in vitro exposure at 37 C and 5% CO2 before RNA extraction/qRT-PCR; no repeat dosing or time-course |
| ST33 | *A. muciniphila* ATCC BAA-835; clade not reported in the article text. | *Akk* was cultured anaerobically in BHI to OD600 0.5-0.6; cells were removed at 8000 x g for 20 min at 4 degC, supernatant filtered through 0.45 um, concentrated by 100-kDa ultrafiltration, filtered through 0.22 um, and OMVs pelleted at 150,000 x g for 1.5 h at 4 degC. siAtox1 (12 ug) and/or ES (1 mg) were loaded into 200 ug OMV by electroporation (400 V, 125 uF), followed by 50-kDa ultrafiltration and three PBS washes. | TEM, DLS/Zetasizer size and PDI, zeta potential, STEM-EDX elemental mapping, Coomassie staining, LC-MS/MS proteomics, 4/37 degC stability, Cy5/gel-based siRNA encapsulation, HPLC ES encapsulation/release. Reported sizes were about 100 nm for native OMV and 164-212 nm after loading; siRNA encapsulation was about 70%, ES encapsulation about 12.3%, with about 40% ES release over 12 h. | OMV protein mass was the in vivo dose metric: 60 ug OMV protein/mouse was used for systemic therapy and biodistribution; the preparation loaded 12 ug siAtox1 and 1 mg ES per 200 ug OMV. BMDC immune assays used 20 ug/mL OMV. | Tail-vein injection. In 4T1 mice, 100 uL injections were given on days 4, 7, 10, 14 and 18 after tumour inoculation. In the CT26-Luc orthotopic model, injections were given on days 7, 10, 13, 16 and 18 after tumour implantation. Biodistribution imaging was performed 24 h after tail-vein injection. |
| ST34 | Methods-level fields require verification against the cited full text and supplementary files. | Methods-level fields require verification against the cited full text and supplementary files. | Methods-level fields require verification against the cited full text and supplementary files. | Methods-level fields require verification against the cited full text and supplementary files. | In vitro exposure; verify concentration and exposure schedule in PMC11599736. |
| ST35 | *A. muciniphila* MucT ATCC BAA-835 and *F. prausnitzii* A2-165 from DSMZ. | 11,000 x g for 20 min; 0.22 micrometre filtration; 200,000 x g for 2 h; PBS resuspension; storage at -80 C. | TEM only. | EVs 1 and 50 micrograms/mL; bacteria MOI 1 and 10. | Direct in vitro exposure for 24 h. |
| ST37 | Methods-level fields require verification against the cited full text and supplementary files. | Methods-level fields require verification against the cited full text and supplementary files. | Methods-level fields require verification against the cited full text and supplementary files. | Methods-level fields require verification against the cited full text and supplementary files. | Intravenous administration; frequency and timing require verification in PMC11362604. |
| ST38 | *A. muciniphila* ATCC BAA-835 from a Chinese culture collection; clade not reported. | BHI plus 0.125% mucin; 10,000 x g for 30 min; 0.22 micrometre filtration; 100,000 x g for 70 min; PBS resuspension. | TEM, NTA, OmpA/OmpC immunoblotting, Bradford protein assay and Coomassie staining. | OMVs 100 micrograms/mL in vitro; OMVs or recombinant Amuc_1434 150 micrograms/kg/day intraperitoneally for 21 days. | In vitro for 48 h; intraperitoneal treatment in the reported xenograft model. |
| ST39 | Methods-level fields require verification against the cited full text and supplementary files. | Engineered hybrid assembly; verify component ratios and process controls in PMC10628780. | Methods-level fields require verification against the cited full text and supplementary files. | Methods-level fields require verification against the cited full text and supplementary files. | Peritumoral administration was reported; schedule and dose require full-text verification. |
| ST40 | *A. muciniphila* KCTC 15667; clade not reported. | 7,000 x g for 30 min; 0.45 and 0.22 micrometre filtration; 15% PEG-6000 overnight; 8,000 x g for 30 min; PBS resuspension and 0.22 micrometre final filtration. | TEM and NTA; no bacterial-EV positive/negative-marker panel or orthogonal purification. | Live bacteria 10^9 CFU/mL; EV-enriched preparation 10^10 particles/mL. | Oral gavage over a 21-day experiment; frequency was not clearly stated in the methods text. |
| ST41 | *A. muciniphila* reported; strain details not verified from the available source | EV isolation reported in the source; exact workflow retained for full-method verification | Particle and cargo characterization reported; release-level sufficiency requires full-method audit | Dose details not verified from the available source | Route and schedule not verified from the available source |
| ST42 | *A. muciniphila* supplied by Nanchang University; strain/clade identifier not reported. | BHI culture; 12,000 rcf for 20 min; sequential 2, 1.2, 0.45 and 0.22 micrometre filtration; 150,000 x g for 2 h; PBS resuspension; storage at -80 C. | TEM, NTA particle size/concentration and BCA protein assay. | 20 micrograms per mouse per day; DiR-labelled material for imaging. | Oral gavage after MK-801 exposure and washout; exact treatment duration was not stated in the methods text. Imaging at 24 h. |
| ST43 | *Akkermansia muciniphila* ATCC BAA-835 supplied the EV preparation used for cargo discovery; recombinant proteins were separate interventions. | Bacteria were grown in BHI supplemented with mucin and cysteine. Culture supernatants underwent filtration, concentration and OptiPrep purification; the subsequent osteoporosis treatment experiments administered recombinant proteins rather than intact EVs. | TEM and nanoparticle tracking characterized the isolated vesicles. EV proteomic analysis supported candidate-cargo identification; human plasma EV-associated protein measurements formed a separate observational component. | Recombinant proteins: 1 µg/mL in cells; 4 µg/mouse. These are not intact-EV doses. | Protein i.v. weekly for 8 weeks, starting one week after ovariectomy. |
| ST44 | *Akkermansia muciniphila* ATCC BAA-835 was used; isolated EV cell experiments must be distinguished from the live-bacterium SAMP8 mouse arm. | Anaerobic cultures in soy-peptone medium were harvested at OD600 0.65. Supernatants were filtered through 0.45- and 0.22-µm membranes and ultracentrifuged at 140,000g for two hours. | EV protein content was measured by BCA. Detailed particle-level purity and identity evidence is not established by the EV extraction description reviewed here; protein concentration alone does not demonstrate purity. | EV protein 0–10 µg/mL; dexamethasone 5 µM. | Cell exposure 24 h for atrophy/protein-synthesis assays. No animal EV dosing. |
| ST46 | *A. muciniphila* ATCC BAA-835 OMVs were combined with OMVs from engineered *E. coli* BL21(DE3) expressing the ClyA–HA–CD326 construct. | Filtered, ultrafiltered supernatants underwent 150,000g centrifugation for two hours. *E. coli* and *AKK* OMVs were fused at a 1:2 protein ratio by sonication/extrusion; tetrathiomolybdate was loaded by electroporation. | TEM, dynamic light scattering, zeta potential and polydispersity characterized the hybrid. FRET assessed membrane mixing; protein profiles, drug loading/release and stability measurements further described the engineered product. | 60 µg hybrid OMV protein containing 25 µg tetrathiomolybdate/mouse. | Tail-vein injections on days 4, 7, 10, 14 and 18; tumor endpoint day 20. |
| ST48 | The publisher abstract identifies *A. muciniphila* MucT; an accession-level strain confirmation requires the unavailable full methods for this study. | The abstract describes isolated EVs alongside live bacteria, UV-killed bacteria and cell-free supernatant. Isolation, washing, purification and storage details could not be verified without the full text. | The accessible abstract reports spherical EVs measuring approximately 50–200 nm by TEM. Additional concentration, purity and compositional assessments are unverified, not assumed to have been omitted. | EVs 20 µg/mL; toxin-containing supernatant 500 µg/mL. | Cell treatment; exposure duration unavailable in abstract. |
| ST50 | *A. muciniphila* DSM 22959 provided native OMVs; other bacterial cultures appeared in separate uptake and interspecies-interaction experiments within the study. | *AKK* was cultured anaerobically in BHI for four to seven days. Supernatants underwent 0.45-µm filtration and ultracentrifugation at 170,000g for 60 minutes; oral dosing was normalized to OMV protein. | Nanoparticle tracking characterized particle size and concentration, including supplementary simulated-gastric-fluid stability experiments. Cy5.5 labelling was assessed by flow cytometry and confocal microscopy before uptake studies. | 20 µg OMV protein in 100 µL per gavage. | Colitis: daily for 5 days. Tumors: OMVs before anti-PD-1 cycles (100 µg i.p., three doses every 3 days). |
| ST51 | *A. muciniphila* ATCC BAA-835 was tested as live bacteria, heat-inactivated bacteria, supernatant and a chemically extracted membrane-vesicle preparation. | Bacteria were grown in supplemented BHI. Membrane vesicles were extracted from bacterial pellets with EDTA/sodium deoxycholate and sequential centrifugation, rather than purified solely from spontaneously released culture-supernatant vesicles. | TEM and SDS-PAGE were used to examine the membrane-vesicle preparation. Its extraction chemistry distinguishes it from native secreted EVs and should remain explicit when comparing composition or bioactivity across studies. | 50 or 100 µg/mL OMVs. | 24-h cell exposure. |
| ST53 | *A. muciniphila* ATCC BAA-835 supplied the pure-*AKK* EV arm; child/elderly mixed-microbiota EVs and *E. coli* EVs were separate comparators. | Bacteria were cultured for four days in mucin/cysteine-supplemented BHI. Supernatants underwent 0.22-µm filtration, ultrafiltration and OptiPrep purification; preparations were normalized by particle number for cellular and animal dosing. | TEM, nanoparticle tracking and dynamic light scattering characterized EV preparations, with a culture-based sterility check. Supplementary data compared particle counts across preparations and batches; mixed-microbiota and *AKK* EVs remained separate products. | Oral: 1.2×10^10 particles; i.v.: 3×10^9 particles. Cells: 6×10^8 particles/mL. | Twice weekly for 2 months; route-specific tracking at 1 h. |
| ST54 | *A. muciniphila* ATCC BAA-835 was used for both the native EV preparation and a separate live-bacterium comparison in healthy mice. | Bacteria were grown in mucin-supplemented BHI to OD600 1. Culture supernatants were filtered and ultracentrifuged at 200,000g for two hours; the source's filtration-unit inconsistency should not be silently corrected. | TEM was used to examine the isolated EV preparation, and dosing was expressed by protein amount. These measurements should not be interpreted as a complete demonstration of vesicle purity or cargo identity. | 10 µg EV protein in 200 µL/mouse; cells 10 µg per treatment. | Oral daily for 4 weeks; cells 24 h. |
| ST55 | *A. muciniphila* ATCC BAA-835 supplied the EV pilot; the metformin and fecal-transfer experiments were distinct parts of the article. | Bacteria were cultured anaerobically at 37°C for seven days in BHI with resazurin/cysteine. After centrifugation and filtration, EVs were pelleted at 150,000g for three hours and resuspended in PBS. | The EV methods describe culture-supernatant isolation and protein-mass dosing but provide limited preparation-level characterization. Particle size, concentration and purity cannot be reconstructed reliably from this EV pilot's methods alone. | 20 µg EVs/mouse. | Oral daily: methods/results say 5 weeks; Fig. 5 says 9 weeks. |

# Supplementary Table S4 Experimental controls, mechanistic tests, exposure and safety

These are descriptive experimental features, not formal risk-of-bias ratings. Label distribution does not establish delivery of intact vesicles, and pathway inhibition does not establish the necessity of a particular EV cargo. Routine efficacy measurements and short-term tolerability do not constitute a route-specific repeat-dose safety package. Missing information in the retained extraction is not proof that an experiment was not performed.

| **Study** | **Sample size and replication within the study** | **Comparators and preparation controls** | **Mechanistic test** | **Tracking and safety observations** |
| --- | --- | --- | --- | --- |
| ST01 | Distribution n=5-7/group across 3 experiments; permeability assay at least n=6/group and minimum 3 experiments; in vitro assays performed in triplicate | NCD, HFD, NCD+AmEV, HFD+AmEV, untreated Caco-2, LPS, LPS+*E. coli* EV, LPS+AmEV, compound C AMPK-inhibitor conditions Partial: *E. coli* EV and LPS comparators included, but EV-depleted medium, soluble/free-cargo, and cargo-loss controls were not evident in extracted sections | AMPK inhibitor compound C reduced AmEV-induced occludin response; no direct EV-cargo perturbation or vesicle-specific molecule rescue | Cy7-AmEV biodistribution by IVIS/ex vivo imaging; body weight, FITC-dextran permeability, colon length, H&E and glucose tolerance; no dedicated repeated-dose toxicology package |
| ST02 | Experiments performed in triplicate; no animal or human sample-size component | PBS or sucrose vehicle controls, live *A. muciniphila* MOI 10, and three EV dose levels Limited: PBS/sucrose vehicle controls included, but EV-depleted supernatant, heat-inactivated EVs, soluble/free-cargo, RNase/proteinase, live-bacterium depletion or strain/clade comparators were not reported | No causal perturbation beyond comparing live bacteria with EV dose levels; no receptor blockade, cargo knockdown/depletion or rescue experiment | Trypan-blue assay reported no difference in Caco-2 viability after 24 h; no biodistribution, PK, immunogenicity, inflammatory cytokine panel or repeat-dose toxicology |
| ST03 | Thirty male C57BL/6 mice total; six groups with n=5/group; Caco-2 cytokine assay performed in duplicate; qPCR/cell experiments reported with standard controls | ND+PBS, ND+live *A. muciniphila*, ND+EVs, HFD+PBS, HFD+live *A. muciniphila*, HFD+EVs; in vitro PBS control, live *A. muciniphila* and EV-treated Caco-2 cells Limited: PBS vehicle and live-bacterium comparator included, but EV-depleted supernatant, heat-inactivated EVs, soluble/free-cargo, RNase/proteinase, cargo-loss/rescue or strain/clade comparators were not reported | No mechanistic perturbation beyond live bacterium versus EV comparison and diet and cell-model comparisons; no receptor blockade, cargo knockdown/depletion or rescue experiment | Cell viability after treatment was checked and >98% survival was reported for Caco-2 cells; body weight, food/water intake, blood glucose/lipids and H&E were monitored; no biodistribution, PK, immunogenicity, systemic cytokine panel in mice or repeat-dose toxicology package |
| ST04 | Thirty-five male C57BL/6 mice; five groups with n=7/group; serological/histological tests performed in duplicate | ND+PBS, HFD+PBS, HFD+live *A. muciniphila*, HFD+pasteurized *A. muciniphila* and HFD+EVs Limited: PBS, live-cell and pasteurized-cell comparators included, but EV-depleted supernatant, heat-inactivated EVs, soluble/free-cargo, RNase/proteinase, cargo-loss/rescue or strain/clade comparators were not reported | No cargo or receptor perturbation; mechanistic inference is based on comparative phenotype and gene-expression changes across live, pasteurized and EV treatments | Plasma metabolic enzymes/lipids/cytokines, eWAT cytokines, body weight, food intake and tissue histology were monitored; no biodistribution, PK, immunogenicity, dose-ranging safety or GLP repeat-dose toxicology package |
| ST05 | Twenty-five male C57BL/6 mice; five groups with n=5/group; LX-2 experiments performed in duplicate and repeated at least three times; fecal qPCR performed in triplicate | ND healthy control, HFD/CCl4+PBS vehicle, HFD/CCl4+live *A. muciniphila*, HFD/CCl4+pasteurized *A. muciniphila*, HFD/CCl4+EVs; quiescent and LPS-activated LX-2 controls Limited: PBS, live-cell and pasteurized-cell comparators included, but EV-depleted supernatant, heat-inactivated EVs, soluble/free-cargo, RNase/proteinase, cargo-loss/rescue or strain/clade comparators were not reported | Dose-response-like LX-2 testing identified EV 50 ug/mL as most active and oral EVs improved multi-tissue markers; no cargo/receptor perturbation, loss-of-function or rescue experiment | Serum liver enzymes, glucose/lipids and cytokines, body/liver weight, histology and multi-tissue qRT-PCR were monitored; no EV biodistribution, PK, immunogenicity, dose-ranging safety or repeat-dose toxicology package |
| ST06 | Twenty-five male C57BL/6 mice; five groups with n=5/group; LX-2 experiments performed in duplicate and repeated at least three times | Standard-diet healthy control, HFD/CCl4+PBS vehicle, HFD/CCl4+live *A. muciniphila*, HFD/CCl4+pasteurized *A. muciniphila* and HFD/CCl4+EVs; untreated and LPS-activated LX-2 controls Limited: PBS, live-cell and pasteurized-cell comparators included, but EV-depleted supernatant, heat-inactivated EVs, soluble/free-cargo, RNase/proteinase, cargo-loss/rescue or strain/clade comparators were not reported | Inflammatory-axis perturbation is inferential: EV dose testing in LX-2 and in vivo TLR5/TLR9/PPAR changes support pathway involvement, but no receptor/cargo loss-of-function or rescue experiment was performed | Liver/adipose cytokines, serum aminotransferases, histopathology/histometry and tissue qRT-PCR were monitored; no EV biodistribution, PK, immunogenicity, dose-ranging safety or repeat-dose toxicology package |
| ST07 | Tumor treatment n=5/group; Ki67 and liver/kidney H&E n=3/group; CD8 flow cytometry n=5/group; macrophage qPCR n=3/group; CCK-8/wound assays commonly n=4/group; colony formation n=3/group | Normal saline/PBS solvent controls; CM from solvent-treated macrophages; untreated/solvent in vitro controls; normal-cell tolerance panel No EV-depleted fraction, soluble/free-cargo, heat/lysis, RNase/proteinase, cargo-loss or rescue control evident in extracted full text | Immune association supported by CD8+ T-cell activation, M1/M2 macrophage profiling and macrophage-conditioned-medium transfer; no CD8/macrophage depletion, receptor blockade, or EV-cargo perturbation/rescue | Body mass, food and water consumption, liver/kidney H&E, and normal-cell CCK-8 viability; no dedicated biodistribution, PK, immunogenicity, cytokine-release or repeat-dose toxicology package |
| ST08 | Animal PE groups reported n=6/group; biodistribution n=3/group; placental transcriptome n=3/group; fertility assay n=12/group; HTR-8/SVneo proliferation and western blot n=3/group, wound-healing n=6/group | CTRL PBS, PLN L-NAME+PBS, AmLN live *A. muciniphila*+L-NAME and AmEVLN AmEVs+L-NAME; dye/PBS procedural controls for uptake and biodistribution; in vitro H/R control plus AmEV dose groups PBS vehicle, live-bacterium comparator and dye/procedural controls were included; EV-depleted supernatant, heat-inactivated EVs, soluble/free-cargo, RNase/proteinase, cargo-loss/rescue and receptor/pathway blockade controls were not reported | Supports activity of the administered preparation with label distribution, uptake-associated signal, disease responses and EGFR-PI3K-AKT activation, but mechanistic causality remains incomplete without EGFR/PI3K inhibition, cargo loss-of-function or rescue experiments | Natural fertility assay reported no toxic impact on fertility or offspring after preconception Am/AmEV gavage; kidney histology was monitored in PE experiments; no quantitative PK, immunogenicity panel, biodistribution time-course beyond 5 h, dose-ranging safety or GLP repeat-dose toxicology |
| ST09 | Four mouse groups; figure captions report n=6 for clinical/inflammatory indicators and n=5 for permeability/16S readouts; RAW264.7 assays shown as n=5 | Control+PBS, DSS+PBS, DSS+*Akk* live bacteria and DSS+*Akk* EVs; in vitro control, LPS, LPS+*Akk* and LPS+*Akk* EV dose groups Limited: PBS and live-bacterium comparator included, but EV-depleted supernatant, heat-inactivated EVs, soluble/free-cargo, RNase/proteinase, cargo-loss/rescue or strain/clade comparators were not reported | Cellular uptake plus dose-range anti-inflammatory testing supports EV activity, but no receptor/cargo loss-of-function, blockade or rescue experiment was performed | Body weight, DAI, serum inflammatory/permeability markers and histology were monitored; no EV biodistribution beyond macrophage uptake, PK, immunogenicity, dose-ranging safety or GLP repeat-dose toxicology |
| ST10 | Healthy-donor PBMC-derived cultures; donor count not reported. Replication wording is internally inconsistent. | PBS, LPS, dexamethasone and live *A. muciniphila*. No EV-depleted supernatant, matched process control or soluble-cargo control. | No depletion, receptor perturbation or rescue experiment. | Cell-only viability and phenotype findings; no organism-level safety assessment. |
| ST11 | Random assignment; animal analyses generally n=6; minimum four replicates for experiments. | Vehicle, two dose levels, normotensive strain and ex vivo acetylcholine response. No EV-depleted supernatant or soluble-cargo control. | Immune associations only; Treg/Th17 necessity not tested. | Body weight and observation of serious adverse reactions; no complement, coagulation, organ histology or immunogenicity package. |
| ST12 | Randomized five groups, n=10/group; experiments reported as repeated three times; behavioural blinding not reported. | Sham, disease model, live *A. muciniphila* and PLX5622 microglial-ablation group. No sonication-process control, membrane-fragment control or soluble-cargo control. | Microglial ablation supports cellular dependency for selected outcomes; no TLR2/4 perturbation or product-level mediator rescue. | No dedicated toxicology, immunogenicity or exposure assessment. |
| ST13 | Randomized groups, n=5/group; cytokine assays were duplicated and performed blind; no sample-size justification. | Normal diet, HFD, HFD/CCl4, live bacteria and pasteurized bacteria. No EV-depleted supernatant, process control or soluble-cargo control. | No causal perturbation. | No dedicated safety, PK or biodistribution assessment. |
| ST14 | All experiments reported as performed in triplicate; no animal sample size because no in vivo study was performed | Control Caco-2 cells, LPS-induced cells and LPS plus Am-EV dose groups (1, 25, 50 ug/mL); no live *AKK* or EV-depleted/heat-inactivated EV comparator Limited to dose-response and untreated/LPS controls; no EV-depleted supernatant, heat-inactivated EVs, soluble/free-cargo, RNase/proteinase, cargo-loss/rescue, receptor/pathway inhibitor or strain/clade comparator | RNA-seq and western blot support MAPK-associated anti-inflammatory signalling, but causal mechanism remains unproven without pathway inhibition, knockdown, cargo depletion or rescue | MTT viability suggested no cytotoxicity across tested Am-EV concentrations and possible Caco-2 proliferation support; no animal safety, biodistribution, PK, immunogenicity or formal toxicology data |
| ST15 | Duplicate cytokine assay and triplicate PCR reactions; biological-replicate basis not clearly specified. | PBS and viable *A. muciniphila*. No EV-depleted supernatant, matched process control or soluble-cargo control. | No mediator perturbation or rescue. | Trypan-blue viability only. |
| ST16 | Milk microbiota association n=8 healthy and n=8 mastitis cows; cow supplementation n=5/group; MAC-T CCK-8 n=5 and cytokine/WB readouts generally n=3; mouse mastitis histology/cytokine readouts report n=3 | Healthy/mastitis cow groups, Healthy/Mastitis/Mastitis+*Akk* cow supplementation, MAC-T blank/LPS/LPS+LA/LPS+PA/AOMV dose groups, vehicle versus GW4869-treated *A. muciniphila*, mouse PBS control/LPS/AOMV+LPS groups; DiD-labelled AOMV tracing Includes live and pasteurized bacterial comparators, isolated AOMVs and GW4869 secretion-inhibition condition; lacks EV-depleted supernatant, heat-inactivated AOMV, soluble/free-cargo, RNase/proteinase, cargo-loss/rescue, TLR4 blockade/rescue or strain/clade comparator | Causality is stronger than simple association because GW4869 reduced AOMV output and attenuated live-*Akk* effects, and isolated AOMVs reproduced anti-inflammatory phenotypes; however, pathway/cargo causality remains incomplete without receptor inhibition, cargo depletion or rescue | Cell viability and mouse histology/cytokines were monitored; preliminary cow SCC/CMT response was tracked; no dose-ranging animal safety, quantitative PK, immunogenicity, clinical chemistry, biodistribution time-course beyond 12 h or GLP repeat-dose toxicology |
| ST17 | Figure legends report fecal *Akk* n=8, tumor *Akk* n=6, body weight/DAI/intestine length n=9, tumor burden n=6, Ki67/histopathology n=5-6, CRC cell proliferation/colony formation n=5-10 and OMV proteomics from three independently prepared batches; human tumor analysis included up to n=65 with tumor-volume correlation n=33 | CAC, CAC+Stress, CAC+Stress+live *A. muciniphila* and CAC+Stress+OMV groups; basal medium versus *A. muciniphila* supernatant exploratory cell comparison; CRC cell lines with/without OMVs plus normal NCM460 selectivity comparison Includes live-bacterium and supernatant comparators plus normal-cell selectivity testing; lacks EV-depleted supernatant, heat-inactivated OMVs, soluble/free-cargo, RNase/proteinase, cargo-depletion, candidate-protein loss/rescue, receptor blockade or strain/clade comparator | Oral OMV efficacy and CRC-cell uptake support an OMV-mediated antitumor contribution, but cargo/receptor causality remains unproven because no loss-of-function, rescue or blockade experiment was reported | Body weight/DAI and normal NCM460 viability were monitored and OMV SGF/SIF stability was assessed; no quantitative biodistribution, PK, immunogenicity, clinical chemistry, dose-ranging safety or GLP repeat-dose toxicology package |
| ST18 | In vitro growth assays n=3 biological replicates with at least two independent experiments; GF colonization experiments typically n=4/group; ASF gnotobiotic competition n=3; EV-induced IgA assay included n=4 colonized controls and n=8 untreated/EV-treated GF groups; human clade survey included 890 healthy Korean subjects plus global adult 16S datasets | Media/GF fecal controls, AmI-derived EVs versus AmII-derived EVs, heat-inactivated and proteinase K-treated supernatants, <30 kDa and >100 kDa fractions, SEC fractions, WT versus JH-/- mice, and untreated GF controls Substantial fractionation controls: heat, proteinase K, molecular-weight cutoff, SEC fractions, fecal soluble fractions and clade-matched EV controls; however, purified Rhs/cargo-loss or EV-biogenesis mutant rescue was not evident in extracted XML | Causality supported by EV dose-response, disk diffusion, luminal EV fraction activity and JH-/- loss of EV-induced early colonization advantage; no direct genetic deletion or rescue of candidate Rhs cargo/EV biogenesis was reported in extracted XML | Safety/PK/BD not a therapeutic endpoint; GF/gnotobiotic colonization was monitored by fecal qPCR and IgA binding, but no toxicology, biodistribution or repeat-dose safety package was provided |
| ST19 | Animal groups n=15/group, with n=10 for body weight/behavior/molecular experiments and n=5 for brain IF/IHC; figure-level assays commonly n=6-10 for microbiota/metabolomics/cytokines and n=4-5 for immunostaining; HT-22 assays used cell-level experimental replication not fully detailed in XML | Normal control, DSS model/PBS gavage, AmEV-treated group; LPS-only HT-22 model; recombinant Amuc 1100 comparator; TLR2 inhibitor C29 and tryptophan hydroxylase inhibitor PCPA in cellular mechanism tests Limited: PBS/model controls and Amuc 1100/inhibitor tests included, but EV-depleted medium, soluble/free-cargo, heat/lysis, RNase/proteinase, cargo-loss/rescue or strain/clade comparator controls were not evident in extracted XML | Partial: PKH26 uptake, AmEV proteomics, Amuc 1100 rescue, C29/TLR2 and PCPA inhibitor trends support a tryptophan/5-HT mechanism, but no in vivo Tph2/Tlr2 knockout, Amuc 1100-deficient EV, microbiota-transfer or vagotomy causality tests | PKH26 biodistribution to brain/colon/liver/spleen at 6 h, body weight, colitis indices and tissue histology/IF; no dedicated PK quantification, dose-ranging, immunogenicity or repeat-dose toxicology package |
| ST20 | Six main groups n=6/group; Treg-depletion figures n=6/group; at least 50,000 flow-cytometry events acquired per sample; randomization and blinded testing/analysis reported | Control, untreated T1DM, heat-inactivated *A. muciniphila* (*AKK*.m), AmEV low/medium/high doses, Vehicle+IgG, AmEV-H+IgG, Vehicle+anti-CD25 and AmEV-H+anti-CD25 Partial: heat-inactivated whole-bacterium comparator and dose-response included, but EV-depleted fraction, soluble/free-cargo, heat/lysis, RNase/proteinase, cargo-loss/rescue or strain/clade comparator controls were not evident in extracted XML | Anti-CD25-mediated Treg depletion supports Treg-dependent contribution; no active EV cargo, receptor, or EV-biogenesis perturbation/rescue was reported in extracted XML | Daily health monitoring, humane endpoints, body weight, liver ALT/AST/TG and hepatic H&E; no biodistribution, PK, immunogenicity, dose-limiting toxicity or repeat-dose GLP-style package |
| ST21 | H22 antitumor and serum-biochemistry panels report n=6/group; PDO validation n=3; additional PDX and figure-specific n values not reported from the article text used for this audit. | Control, BMV, siRNA@BMV/SB, siRNA-MOF/SM and siRNA-MOF@BMV/SMB; other probiotic BMVs screened; Durvalumab anti-PD-L1 comparison in PDX setting; ferroptosis/necroptosis/pyroptosis inhibitor assays; GdCl3 macrophage depletion. Partial: extrusion, centrifugation and saline washing were used after engineering, and SM/SB/BMV comparators help separate BMV coating from siRNA-MOF effects; residual free siRNA, MOF, dye/tracer and soluble bacterial components were not fully quantified in the extracted XML. | RNA-seq/GSEA, inhibitor assays and marker analysis support pyroptosis rather than ferroptosis/necroptosis as the dominant cell-death route; NLRP3/Caspase-1/GSDMD activation is reported. Macrophage depletion and cGAS-STING/IFN-I readouts support an innate-immune contribution, but epigenetic proof of trained immunity remains incomplete. | Cy5.5-labeled SMB IV imaging showed hepatic/tumor accumulation within 2 h and retention at 24 h, with limited off-target signal aside from renal clearance; body weight, H&E and serum ALT/AST/ALB/ALP in healthy non-tumor-bearing mice were reported, but repeat-dose GLP-style toxicology was not provided. |
| ST22 | Exact animal group n values and panel-specific replication require PDF/supplement/source-data verification; extracted text reports n values mainly in figure legends/supplementary information. | PBS/Cy5 controls, native *AKK*-EVs, BT-*AKK*-EVs, miR-21-5p inhibitor and negative-control inhibitor groups. Partial: engineered versus native EVs and miR-21-5p inhibitor arms support cargo contribution, but EV-depleted supernatant, free miRNA, density-gradient/SEC and cargo-loss/rescue controls should be verified. | miR-21-5p inhibitor experiments in osteogenic and osteoclast assays provide cargo-perturbation support; PI3K-AKT signaling is pathway-associated rather than fully rescued genetically. | Cy5 biodistribution across organs and leg bones; histopathology of major organs and liver/kidney toxicity testing were reported as favorable, but no formal GLP toxicology or PK package. |
| ST23 | n=5 mice/group in DSS/EV figures; duplicate experiments reported for therapeutic-effect evaluation | Water control, DSS alone, DSS + live *A. muciniphila* 5 x 10^8 CFU/mouse, DSS + AmEV; in vitro LPS and *E. coli* EV comparators Limited: EV compared with live bacteria and *E. coli* EV, but no EV-depleted fraction, soluble-cargo control, or cargo-loss control reported in extracted main text | No molecular cargo perturbation; evidence is based on taxonomic EV shifts, in vitro cytokine modulation, and DSS-colitis phenotype rescue | Body weight, colon length, DAI, H&E/histological activity index; no dedicated PK, biodistribution, or repeat-dose toxicology package |
| ST24 | Thirty mice randomized to five groups, n=6/group; selected safety outcomes used n=3-5. | Vehicle, DNFB model, dexamethasone, route comparison and free-dye tracking comparator. No EV-depleted supernatant or miR-21-5p-depleted administered preparation. | miR-21-5p mimic/inhibitor and TLR4 reporter evidence; no product-level cargo loss and rescue. | Selected histology and serum ALT/AST/creatinine/urea; small outcome-specific n and no repeat-dose immunogenicity. |
| ST25 | n=5/group for animal outcomes; in vitro experiments at least three replicates. | Air, smoke, live *A. muciniphila* and recombinant P9; labelled-PBS control described for uptake workflow. No EV-depleted supernatant or P9-depleted EV preparation. | Recombinant P9 sufficiency tested; product-level P9 necessity and rescue not tested. | Body and prostate weights; no dedicated route-specific toxicology, immunogenicity or coagulation/complement assessment. |
| ST26 | Clinical n=30; fractionation cognitive tests n=10 mice/group; DiD brain biodistribution n=5; OMV/ILA oral intervention n=9 mice/group; intranasal OMV intervention n=10 mice/group; hippocampal IF n=5; neuroinflammatory mRNA/protein n=3; metabolic cell assays n=6; HMC3/SH-SY5Y in vitro assays as reported in figure panels | Nonsmoking control/MCI smoker groups; filtered-air aged controls; PBS vehicle; FMT control donors; *E. coli* negative-control conditioned medium; *A. muciniphila*, *A. muciniphila*-CM fractions, proteinase K-digested CM, purified ILA; CH223191 AhR inhibitor and vehicle Includes PBS/vehicle, filtered-air/smoke, FMT donor controls, *E. coli* negative control, purified ILA, CM fractionation and AhR inhibitor; lacks EV-depleted supernatant, heat-inactivated OMVs, ILA-depleted OMVs, OMV cargo-loss/rescue and in vivo AhR genetic controls | Strong mechanistic chain from human association to FMT, OMV brain delivery, ILA cargo, microglial uptake and AhR-inhibitor loss of effect in vitro, but in vivo *A. muciniphila*-specific depletion, ILA-depleted OMV rescue and microglia-specific AhR knockout were not performed | Behavior, body/brain readouts and cell viability/neuronal injury markers were monitored; no formal OMV dose-ranging safety, repeat-dose toxicology, immunogenicity, clinical chemistry, quantitative PK or biodistribution time-course beyond 24 h brain imaging |
| ST27 | Outcome figures generally n=5/group; omics n=3/group; experiments reported as repeated three times. | Saline, OVA model, native OMVs, PMB-treated OMVs and PMB alone. PMB-only control addressed residual PMB; no EV-depleted supernatant or matched non-vesicular process control. | PMB modification and macrophage readouts were compared; AP-1/PI3K/AKT pathway necessity was not tested. | Local lung histology and PMB-only comparison; no dedicated repeat-dose immunogenicity or systemic toxicology. |
| ST28 | Randomized groups generally n=3 for accumulation/efficacy/safety and n=6 for survival; no blinding or sample-size justification reported. | PBS, free siRNA, liposome-loaded siRNA, protease-inhibitor-treated AMV construct and alginate-microgel formulation. Free siRNA and unbound-cargo removal were addressed; no scrambled-siRNA control identified. | PD-L1 target engagement and COPI/mucus-penetration perturbation were tested; carrier-intact exposure was not established. | Thirty-day body weight, serum ALT/AST/ALP/LDH/BUN, major-organ histology and mucus assessment, generally n=3/group. |
| ST29 | Human cohort 1 n=62 fecal samples; cohort 2 n=92 with serum n=79 and NP tissue n=29; MR 41,669 IVDD cases and 294,770 controls with 59 SNP IVs; main puncture figures n=8/group; aging readouts n=8-16/group; DiR tracing n=3/group; proteomics and cell assays n=3/group | Vehicle/PBS, non-punctured or baseline controls, *E. coli*, *E. coli*-EVs, *Akk*-GW4869, DMSO/vehicle, TNF-alpha-only cells, five recombinant candidate proteins and untreated age controls Partial: *E. coli* and *E. coli*-EVs address strain/EV specificity, GW4869 tests EV-secretion dependence, and recombinant B2UKX5 tests sufficiency; soluble/free-cargo depletion, B2UKX5-depleted EVs, cargo-loss/rescue and nuclease/protease controls were not evident | EV-dependence supported by GW4869 loss of protection; biodistribution supported by DiR-labeled *Akk*-EVs in discs; cargo nomination by EV/*Akk* proteomics and candidate screen; B2UKX5 sufficiency supported by recombinant protein in cell and mouse IVDD models; no genetic cargo knockout/depletion reported | *Akk*/*Akk*-EVs and B2UKX5 did not elevate systemic cytokines or serum biochemistry in reported models; major organs showed no additional treatment-related toxicity in aging experiments; *E. coli*-EVs induced splenomegaly/cytokines after intravenous dosing; no dedicated PK, biodistribution beyond DiR tracing, immunogenicity or GLP repeat-dose toxicology |
| ST30 | 42 male C57BL/6J mice; after AOM/DSS modeling, control/stress groups n=21 each before intervention; final five intervention groups n=7/group; cell assays performed in HCT-116 and HT-29 lines | Control, Stress, OMVs, Stress+OMVs, Stress+OMVs+Fetuin-A; in vitro ISO +/- OMVs; heat-inactivated *A. muciniphila* OMVs, blank medium processed through the OMV isolation workflow, OMV-depleted culture supernatant and *E. coli*-derived OMVs Relatively strong for active-vesicle specificity: heat-inactivated OMVs, blank medium processed with the same isolation protocol, OMV-depleted supernatant and *E. coli* OMVs did not reproduce active *A. muciniphila* OMV effects in extracted sections | Fetuin-A was nominated by proteomics and database analysis, induced by ISO/CUMS, suppressed by OMVs, and partially restored by exogenous Fetuin-A in stressed OMV-treated mice; upstream OMV cargo and receptor mechanism remain unresolved | Body weight, disease activity index, open-field/tail-suspension behavior and tumor burden reported; OMV uptake assessed in CRC cells; no formal biodistribution, PK, systemic cytokine, immunogenicity or repeat-dose toxicology package identified in extracted XML |
| ST31 | Random-number allocation; four groups, n=10/group; behavioural recordings and analysis blinded. | Vehicle, EV-only and sevoflurane model groups. No EV-depleted supernatant, process control or soluble-cargo control. | No NLRP3 genetic or pharmacological perturbation. | Body weight followed; no dedicated toxicology or immunogenicity. |
| ST32 | Twelve treatment/control groups; each experimental condition tested in two independent biological replicates with technical triplicates; figure data shown as mean +/- SEM | PBS vehicle control, 3% sucrose buffer control for OMVs and BHI medium control for supernatant; active bacterial MOI groups, heat-inactivated bacterial MOI groups, OMV 50/100 ug/mL groups and 7% cell-free-supernatant group Includes live-bacterium, heat-inactivated-bacterium and soluble-supernatant comparators plus vehicle/buffer/medium controls; lacks EV-depleted supernatant, detergent-buffer-only OMV isolation control, heat-inactivated OMVs, size-exclusion/gradient purification comparator, purified cargo, receptor blockade or functional barrier comparator | Supports a direct association between *A. muciniphila* OMV exposure and occludin mRNA induction at 50 ug/mL in Caco-2 cells, but causality is limited because the study did not test cargo, receptor, pathway, protein-level or permeability rescue/loss-of-function mechanisms | No dedicated safety package; no reported Caco-2 viability/cytotoxicity assay, endotoxin/sterility testing, epithelial permeability safety readout, in vivo tolerability, immunogenicity, PK or repeat-dose toxicology |
| ST33 | Subcutaneous 4T1 treatment experiment: n=5 mice/group. Orthotopic CT26-Luc model: n=3 mice/group. Several mechanistic and biodistribution assays were reported with n=3-4 depending on figure panel; verify figure-specific n values before formal meta-extraction. | PBS, siAtox1@OMV, ES@OMV, free ES, free Cy5-siAtox1 for biodistribution, and cisplatin in comparative efficacy/toxicity panels; in vitro comparisons included Atox1 knockdown/KO and drug combinations. Post-electroporation constructs were retained in 50-kDa ultrafiltration tubes and washed three times with PBS; free ES and free Cy5-siAtox1 comparator arms were included. Residual free cargo after washing was not fully quantified beyond loading/encapsulation assays, so this remains a translational control gap. | Atox1 siRNA knockdown was measured by RT-qPCR in tumours and tissues; Atox1 KO cells, ICP-MS copper measurement, LIAS/FDX and cuproptosis/proteotoxic-stress readouts supported copper-homeostasis causality, but the design does not fully disentangle OMV immune stimulation from cargo-driven cuproptosis. | Cy5 biodistribution showed tumour accumulation with liver signal; dose optimization noted limited efficacy at 20 ug and tolerability concerns at 100 ug (piloerection/reduced food intake), leading to 60 ug selection. Body weight, serum IL-6/IFN-alpha, histology and cisplatin comparison were used for short-term safety observations; long-term immunotoxicity, repeat-dose biodistribution and clinical-grade release criteria remain absent. |
| ST34 | Methods-level fields require verification against the cited full text and supplementary files. | Methods-level fields require verification against the cited full text and supplementary files. not reported beyond the available abstract or article text. | not reported beyond the available abstract or article text. | No in vivo PK, biodistribution, or safety inference is permitted from this model. |
| ST35 | Experiments performed in triplicate; biological independence not further specified. | PBS, two bacterial species and corresponding live-bacteria exposures. No EV-depleted supernatant or process control. | No perturbation or rescue. | Cell-line experiment only. |
| ST37 | Methods-level fields require verification against the cited full text and supplementary files. Shared animal tissues with ST11; not an independent cohort. | SHR and WKY strain comparison; exact vehicle and baseline controls require full-text verification. not reported beyond the available abstract or article text. | not reported beyond the available abstract or article text. | PK, intact-vesicle biodistribution, and systemic safety remain insufficiently established. |
| ST38 | In vitro triplicates; randomized mouse groups n=5/group; blinding and sample-size justification not reported. | PBS and recombinant Amuc_1434; no established carrier or immune-checkpoint benchmark. Recombinant protein tested, but OMV cargo depletion and matched process controls were absent. | Recombinant Amuc_1434 sufficiency and co-IP; no product-level cargo necessity or rescue. | Body weight monitoring only; no dedicated route-specific toxicology or immunogenicity. |
| ST39 | Methods-level fields require verification against the cited full text and supplementary files. | Matched empty-hybrid, free-plasmid, single-component, and vehicle controls require complete source-data audit. Free-cargo and single-component attribution controls require full-text verification. | Multicomponent design limits *Akk*-OMV-specific causal attribution. | Local/systemic safety and biodistribution require platform-specific assessment. |
| ST40 | Twenty mice, randomized by body weight to four groups (n=5/group); blinding and sample-size justification not reported. | Normal diet, MASH diet and live *A. muciniphila*; live-cell and particle dose units were not biologically matched. No PEG/process control, EV-depleted fraction or soluble-cargo control. | Transcriptional pathway associations; no loss-of-function experiment. | Body weight, serum ALT/AST and metabolic chemistry; no dedicated EV toxicology or immunogenicity. |
| ST41 | Study-specific sample sizes not verified from the available source | Mechanistic and intervention comparators reported Free B2URF3 or matched cargo controls not verified from the available source | B2URF3/ALDH1B1 perturbation reported; vesicle-versus-mediator rescue hierarchy requires rechecking | No general intact-product vascular PK claim assigned |
| ST42 | Behaviour and biochemical outcomes generally n=5-8; distribution imaging n=3. | Normal and MK-801 model groups; free DiR comparator for imaging. Free dye was compared for imaging; EV-depleted supernatant and soluble-cargo controls were not reported. | No preparation-dependency, cargo-loss or mediator-rescue experiment. | No dedicated repeat-dose toxicology or immunogenicity package; authors acknowledged that PK and dose-range safety were not established. |
| ST43 | Protein bone outcomes: 5–8/group; cells: 3/group. Human BMD: 30 men/41 women; biomarker/age: 51 men/41 women. | Vehicle, sham, Amuc_1426/Amuc_1213; target-gene knockdown controls. | EV cargo discovery was followed by recombinant-protein comparisons and target-gene perturbation in bone-cell experiments. The animal intervention supports Amuc_1473 activity, not equivalence between purified protein and an intact-EV therapeutic product. | Human plasma EV-associated Amuc_1473 was evaluated against bone measures. These associations, and the recombinant-protein animal experiments, do not establish intact-EV biodistribution or therapeutic safety. Rabbit EV immunization served antibody production. |
| ST44 | Cell biological replicate count not explicit in Fig. 6/methods. Live-bacteria groups: 6/6/5 mice. | Untreated/dexamethasone controls; heat-inactivated bacteria in supplementary experiment. | C2C12 differentiation, dexamethasone-induced atrophy and puromycin-based protein-synthesis assays tested EV bioactivity. The experiments support cellular myogenic effects, while the in-vivo sarcopenia findings belong to live bacteria. | The EV intervention was confined to cultured muscle cells. No EV-treated mouse arm was presented; consequently, the live-bacterium animal findings cannot supply EV pharmacokinetic, biodistribution or systemic-safety evidence. |
| ST46 | Tumor growth/weight: 5/group; selected imaging/histology: 3/group. | PBS, free chelator, unloaded targeted hybrid; copper supplementation and anti-PD-1 combinations. | Free chelator, unloaded hybrid and loaded targeted hybrid comparisons probed copper depletion. Metal imaging, metabolomics, immune phenotyping, copper supplementation and anti-PD-1 combinations examined metabolic and immune consequences; native *AKK*-EV efficacy was not isolated. | Cy5.5-labelled hybrid distribution was imaged over 48 hours and in excised organs. Supplementary safety readouts included body weight, organ histology, ALT, AST and BUN; conclusions apply to this hybrid formulation and tested exposure. |
| ST48 | Unavailable in accessible abstract. | Live/UV-killed bacteria (MOI 10), cell-free supernatant, untreated cells. | Caco-2 cells challenged with C. difficile RT001 toxin-containing supernatant were assessed for viability, cytokines and barrier-related genes. Full text is needed to separate EV-specific effects from those of other bacterial preparations. | At the reported EV concentration of 20 µg/mL, cell viability exceeded 90% of untreated controls. This is cellular compatibility evidence, not animal safety or human infection-treatment evidence; additional assays remain unverified. |
| ST50 | Colitis 5/group; mucosal/tumor readouts ≥5; selected in-vitro assays 3. | PBS, healthy/DSS controls, live *AKK* 10^8 CFU; matched anti-PD-1 background. | Bacterial uptake/growth experiments, epithelial assays and dendritic-cell/T-cell co-cultures examined microbiota, mucus/barrier and mucosal immune responses. Mouse studies tested colitis outcomes and anti-PD-1 combination activity, rather than a single established cargo–receptor mechanism. | Cy5.5 signals were evaluated in bacteria, intestinal epithelium and Peyer's patches, including ligated intestinal segments. Such local uptake experiments do not establish oral systemic pharmacokinetics; colitis and tumor efficacy endpoints are not comprehensive toxicology. |
| ST51 | Six wells mentioned for RNA collection; independent biological replicate number not established. | Live/heat-inactivated *AKK*, 7% cell-free supernatant; matched PBS/sucrose/medium controls. | Caco-2 and HepG2 cells were exposed for 24 hours, followed by endocannabinoid-system and PPAR transcript measurements. These expression responses do not establish pathway necessity or functional improvement in a liver-disease model. | Experiments were cellular, with matched preparation-related controls. The reviewed study does not provide an EV-treated animal arm, so its gene-expression results cannot establish animal biodistribution or systemic therapeutic safety. |
| ST53 | Oral bone structure/strength: 8/group; histology 5; dynamic formation 4; cell assays 3 biological replicates. | Sham/OVX vehicle, child/elderly microbiota EVs, *E. coli* EVs and non-EV fractions in relevant experiments. | EV/non-EV comparisons, inhibition-associated experiments and direct BMSC/RAW264.7 treatment examined vesicle involvement in osteogenesis and osteoclastogenesis. OVX mice supplied bone structure, strength and formation/resorption outcomes; no individual *AKK* cargo was established as the sole mediator. | DiR/PKH-labelled EV imaging and EV-antibody staining evaluated tissue distribution, including bone and other organs. Signals at one hour support exposure-related observations, not quantitative intact-vesicle pharmacokinetics or a comprehensive systemic-safety conclusion. |
| ST54 | 30 mice, 10/group; cell experiments in triplicate. | PBS and live *AKK* 10^9 CFU/mouse; cell live-bacteria MOI 100. | Serotonin ELISA and serotonergic-pathway transcripts were measured in colon, hippocampus and Caco-2 cells, alongside inflammatory genes. Tph1 induction without increased cellular serotonin illustrates the distinction between transcript responses and functional pathway output. | Healthy-mouse weight, colonic histology and inflammatory readouts were examined. Survival of intact EVs through the gastrointestinal tract and blood–brain-barrier passage were not directly demonstrated; the study was not a depression-treatment trial. |
| ST55 | EV group 3; HFD control 4. | HFD without EVs; metformin and FMT are separate experiments. | The EV pilot measured weight, glucose, lipids and tissue weights; microbiota changes were described as nonsignificant without displayed data. It does not establish a specific EV cargo mechanism, and metformin/FMT mechanisms should not be transferred. | Body and tissue weights are limited tolerability-related observations, not biodistribution or comprehensive safety. EV treatment duration differs between methods/results and Fig.5, which should be retained as an unresolved exposure-reporting inconsistency. |

# Supplementary Table S5 Risk of bias in animal EV intervention studies

Domains follow SYRCLE: D1 sequence generation; D2 baseline characteristics; D3 allocation concealment; D4 random housing; D5 investigator/caregiver blinding; D6 random outcome assessment; D7 outcome-assessor blinding; D8 incomplete outcome data; D9 selective reporting; D10 other bias. L, low; H, high; U, unclear; NA, not applicable to the scoped animal EV intervention. No total quality score was calculated.

| **Study** | **D1** | **D2** | **D3** | **D4** | **D5** | **D6** | **D7** | **D8** | **D9** | **D10** | **Experimental scope / source limits** |
| --- | --- | --- | --- | --- | --- | --- | --- | --- | --- | --- | --- |
| ST01 | U | U | U | U | U | U | U | U | L | U | HFD mouse permeability experiment; cellular AMPK assays are outside SYRCLE |
| ST02 | NA | NA | NA | NA | NA | NA | NA | NA | NA | NA | Caco-2 cell-line experiment |
| ST03 | U | U | U | U | U | U | U | H | U | U | HFD-induced obesity: weight/adiposity and associated tissue outcomes |
| ST04 | U | U | U | U | U | U | U | L | L | U | Five-week HFD prevention experiment |
| ST05 | U | U | U | U | U | U | L | L | U | U | HFD/CCl4 EV intervention; outcome-assessor judgment specifically for liver/colon histopathology |
| ST06 | U | U | U | U | U | U | L | L | L | U | HFD/CCl4 EV intervention; outcome-assessor judgment specifically for histopathological evaluation |
| ST07 | U | U | U | U | U | U | U | U | L | U | RM-1 syngeneic prostate-tumor mouse experiment |
| ST08 | U | U | U | U | U | U | U | U | L | U | L-NAME pregnancy experiment, distinguishing maternal from fetal outcomes |
| ST09 | U | U | U | U | U | U | U | U | L | U | DSS colitis: disease activity, colon injury and barrier outcomes |
| ST10 | NA | NA | NA | NA | NA | NA | NA | NA | NA | NA | Human-donor dendritic-cell cultures |
| ST11 | U | U | U | U | U | U | U | U | L | U | SHR/WKY blood-pressure and immune experiment; separate ex vivo aortic assay |
| ST12 | U | U | U | U | U | U | U | U | U | U | Intestinal I/R postoperative cognitive dysfunction mouse experiment |
| ST13 | U | U | U | U | U | U | U | U | L | U | HFD and HFD/CCl4 mouse lung endpoints |
| ST14 | NA | NA | NA | NA | NA | NA | NA | NA | NA | NA | LPS-stimulated Caco-2 cell cultures |
| ST15 | NA | NA | NA | NA | NA | NA | NA | NA | NA | NA | STC-1 enteroendocrine cell-line treatment |
| ST16 | U | U | U | U | U | U | U | U | L | U | AOMV mouse mastitis intervention only; dairy-cow bacterial intervention is separate |
| ST17 | U | U | U | U | U | U | L | U | L | U | OMV intervention in stress-associated CAC; outcome-assessor judgment specifically for tissue histopathology |
| ST18 | H | U | U | U | U | U | U | L | L | U | EV-induced IgA and clade-colonization animal experiments |
| ST19 | U | U | U | U | U | U | U | U | L | U | DSS colitis and cognition mouse experiment |
| ST20 | L | U | U | U | U | U | L | L | L | U | STZ T1DM AmEV dose experiment and Treg-depletion extension |
| ST21 | U | U | U | U | U | U | U | U | L | U | Syngeneic H22 treatment experiment; PDX models require separate finalization |
| ST22 | U | U | U | U | U | U | U | L | L | U | OVX bone-loss intervention; separate tracer experiment |
| ST23 | U | U | U | U | U | U | U | U | L | U | DSS colitis EV intervention, not the fecal metagenomic comparison |
| ST24 | U | U | U | U | U | U | U | U | L | U | Mouse AD |
| ST25 | U | U | U | U | U | U | U | U | L | U | Chronic smoke-exposure lung inflammation/fibrosis EV intervention |
| ST26 | U | U | U | U | U | U | U | U | U | U | Smoke/aging-associated cognition, OMV and ILA mouse experiments |
| ST27 | U | U | U | U | U | U | U | U | L | U | OVA asthma with native or polymyxin-B-conjugated OMVs |
| ST28 | U | U | U | U | U | U | U | L | L | U | Orthotopic CT26-Luc tumor inhibition; distribution and safety experiments not automatically pooled |
| ST29 | U | U | U | U | U | U | U | U | L | U | Aging, puncture and bipedal intervertebral-disc mouse models |
| ST30 | L | U | U | U | U | U | U | U | U | U | OMV intervention and Fetuin-A rescue in stress-associated AOM/DSS CRC |
| ST31 | L | U | U | U | U | U | U | U | L | U | Sevoflurane-associated cognitive dysfunction in aged male mice; behavior versus tissue endpoints |
| ST32 | NA | NA | NA | NA | NA | NA | NA | NA | NA | NA | Caco-2 cell-line treatment |
| ST33 | U | U | U | U | U | U | U | U | L | U | 4T1 tumor-growth and CT26 orthotopic tumor experiments; attrition judgment specifically for CT26 bioluminescence |
| ST34 | NA | NA | NA | NA | NA | NA | NA | NA | NA | NA | LS174T indomethacin cell intervention only |
| ST35 | NA | NA | NA | NA | NA | NA | NA | NA | NA | NA | Caco-2 cultures and secondary microarray analysis |
| ST37 | U | U | U | U | U | U | U | U | L | U | SHR/WKY renal follow-up using tissues from the previously published rat cohort |
| ST38 | U | U | U | U | U | U | U | U | U | U | SW480 colorectal xenograft with AKK-OMVs or Amuc_1434 |
| ST39 | U | U | U | U | U | U | U | U | L | H | Native BEV and hybrid-vesicle prophylactic/therapeutic tumor experiments |
| ST40 | U | U | U | U | U | U | U | U | L | U | Murine experiment 3: AKK/AKK-EV validation only; excludes longitudinal rats and Romboutsia arms |
| ST41 | U | U | U | U | U | U | U | U | L | U | Vitamin-D-induced vascular calcification with oral/intravenous Akk-EV and protein interventions |
| ST42 | U | U | U | U | U | U | U | U | U | U | MK-801-associated behavior/neuroinflammation and gut microbiota mouse experiment D9: supplement inspected; reporting completeness unclear. |
| ST43 | NA | NA | NA | NA | NA | NA | NA | NA | NA | NA | EV cargo discovery and human association; recombinant-protein intervention excluded |
| ST44 | NA | NA | NA | NA | NA | NA | NA | NA | NA | NA | EV-treated C2C12 cells only; live-bacterium mouse arm excluded |
| ST46 | U | U | U | U | U | U | U | U | L | U | TM-loaded CD326-targeted E. coli/Akk hybrid OMVs in breast-tumour mice |
| ST48 | NA | NA | NA | NA | NA | NA | NA | NA | NA | NA | EV-treated Caco-2 cells |
| ST50 | U | U | U | U | U | U | U | U | L | U | Akk OMVs in DSS gut-disorder/colitis and CT26 plus anti-PD-1 mouse arms |
| ST51 | NA | NA | NA | NA | NA | NA | NA | NA | NA | NA | EV-treated Caco-2 and HepG2 cells |
| ST53 | U | U | U | U | U | U | U | U | L | U | Akk-EV oral/IV OVX-mouse bone and biodistribution experiments; donor-community and live-bacteria effects excluded |
| ST54 | U | U | U | U | U | U | U | U | L | U | Oral Akk-EVs in healthy male mice; Caco-2 assays not animal evidence |
| ST55 | U | U | U | U | U | U | U | L | U | U | Akk-EV mouse arm |

Two reviewers independently assessed the retained animal EV interventions and agreed on all final domain judgments. Source locators and the assessment history are retained in the accompanying workbook. The table presents the final ratings.

Among 500 domains (410 from retained original reports; 90 from later inclusions), 46 are low, 3 high, 341 unclear and 110 NA. These are domain counts, not study-level ratings. The five exclusions account for 41 unassessed and nine unclear historical entries.

ST36, ST45, ST47, ST49 and ST52 were excluded following two-reviewer eligibility reassessment and are omitted from the current table. Their historical entries remain in the workbook. ST42 remains included with D9 rated unclear.
